# A Calibrated Post-Processing Layer for Tolerance-Compliant Crack-Width Measurement

**DOI:** 10.3390/s26185853

**Published:** 2026-09-15

**Authors:** Ishan Hrishikesh Pradhan, Rodrigo Sarlo

**Affiliations:** 1 Department of Mechanical Engineering, Virginia Tech, Patton Hall, Blacksburg, VA 24060, USA; ish4n@vt.edu; 2 Department of Civil and Environmental Engineering, Virginia Tech, Patton Hall, Blacksburg, VA 24060, USA

**Keywords:** crack-width measurement, measurement reliability, over-segmentation correction, within-tolerance ROI rate, calibration, infrastructure inspection, image segmentation

## Abstract

Crack width governs how inspectors rate and prioritize concrete deterioration, yet automated pipelines are still judged by pixel-overlap scores (IoU, Dice) that do not explicitly target width measurement. We show geometrically that high pixel overlap can coexist with substantial width error and experimentally demonstrate a systematic over-segmentation bias across modern crack-segmentation algorithms. We present Parallel Consensus Fusion (PCF), a deliberately lightweight measurement-reliability layer, applicable to any algorithm whose operating point over-segments, that corrects this bias by intersecting the learned mask with an independently derived contrast-enhanced threshold mask; its few parameters are fixed once per algorithm and domain through a coverage-constrained calibration rather than assumed universal. Evaluated across ten segmentation algorithms on an uncontrolled field set (75 caliper-measured physical regions of interest, ROIs, on 15 cracks, imaged at three distances for 225 image–ROI observations) and an external dataset of 19,098 width-labeled ROIs, PCF behaves as a calibrated over-segmentation corrector whose benefit is predictable from each algorithm’s over-segmentation bias. It raises the *within-tolerance ROI rate*—the share of regions whose measured width falls within an engineering tolerance—for nine of ten algorithms and collapses architecturally diverse algorithms into a single width-accuracy band. We argue that the within-tolerance ROI rate, not overlap, is the metric that matters for inspector-grade crack-width measurement; we evaluate it primarily at a strict ±0.10 mm band, with the looser AASHTO allowable-error bands as robustness checks.

## 1. Introduction

Concrete infrastructure underpins modern transportation systems but is increasingly affected by age-related deterioration and environmental stressors. Cracking is often an early indicator of distress; if unmanaged, it can propagate and compromise serviceability and safety. Because crack width is used to assess severity and plan maintenance, Department of Transportation (DOT) and American Association of State Highway and Transportation Officials (AASHTO) guidance emphasizes width thresholds for assigning condition states and prioritizing interventions. For example, the AASHTO Manual for Bridge Element Inspection defines hairline (≤0.1 mm), small (≈0.2–0.3 mm), and moderate (≥1 mm) crack widths used to drive repair decisions, monitoring schedules, and condition ratings [1,2].

Current workflows frequently rely on manual tools such as crack cards and calipers that require close-up access. This is time-consuming, can increase safety risk, and can introduce variability across inspectors [3]. As agencies expand element-level condition reporting, the need for consistent quantitative field data increases [4]. These needs motivate assisted inspection tools [5] that enable measurements from safer standoff distances (e.g., zoomed photographs) while maintaining near-caliper accuracy.

Numerous machine-learning approaches automate crack detection and segmentation, and reported Intersection over Union (IoU) and Dice scores on benchmark datasets now routinely fall in the 0.80–0.90 range [6,7]. However, pixel-level overlap does not guarantee accurate crack-width measurement. Consider an idealized crack strip of true width *w* whose predicted mask preserves the crack extent but over-segments its boundary uniformly by *d* on each side. In this case, the foreground IoU is w/(w+2d) and the measured width is w+2d, giving $w_{\mathrm{measured}}/w_{\mathrm{true}}=1/\mathrm{IoU}$. Thus, an IoU of 0.80 corresponds to a +25% width overestimate and an IoU of 0.90 to +11%; a ±10% relative width tolerance would require IoU ≥0.91. This relation is specific to uniform lateral over-segmentation and is used here as a geometric illustration rather than as a predictive model; no result in this paper depends on it, since overlap metrics are not used as success criteria anywhere and all reported accuracy is measured against physical width references. When segmentation error is dominated by boundary expansion rather than random misalignment, overlap metrics can therefore obscure systematic width bias. Combined with benchmark datasets that underrepresent low-contrast field conditions, this leaves raw segmentation outputs unreliable for the AASHTO- and DOT-driven width thresholds that govern inspection decisions.

In order to test our over-segmentation hypothesis, we treat crack-width estimation as a measurement problem and target the dominant bias. We introduce Parallel Consensus Fusion (PCF), a calibrated measurement-reliability layer that corrects systematic over-segmentation to recover usable crack width. A brightness-matched image is processed by two independent evidence streams—a learned segmentation mask and a CLAHE-and-threshold mask from a classical branch—whose pixelwise intersection enforces consensus boundaries, trimming over-segmentation while preserving crack continuity. Minimal fixed-parameter filtering removes speckle and shallow attachments, and a two-sided distance transform yields width in millimeters against a per-image scale. PCF is deliberately simple: a drop-in post-processing layer that attaches to any algorithm without retraining. Its substance lies not in architectural novelty but in a stated, testable correction mechanism and a coverage-constrained calibration with explicit, per-domain operating points.

We validate PCF as a crack-width measurement method across ten diverse segmentation algorithms (lightweight CNN, high-resolution CNN, transformer, vision-Mamba, hybrid, and few-shot/low-light), evaluated before and after fusion on two complementary width-labeled datasets: an uncontrolled field set with 225 caliper-referenced image–ROI observations and an external set of 19,098 operator-measured ROIs. Throughout, the primary outcome is the within-tolerance ROI rate: the percentage of all ROIs at which a width is both returned and correct to within the engineering tolerance band. We use inspector-grade as a specific acceptance criterion rather than a slogan—a returned width is inspector-grade when it lies within the AASHTO measurement tolerance (±0.10 mm) of the operator’s caliper reference, the same standard applied to a human inspector.

This work makes three contributions aimed at inspector-grade crack-width measurement:

1. **We demonstrate that pixel overlap is not measurement.** For uniform lateral over-segmentation of an idealized crack strip, we show that $w_{\mathrm{measured}}/w_{\mathrm{true}}=1/\mathrm{IoU}$, illustrating how high overlap can coexist with substantial width error. We then show empirically across ten segmentation algorithms that this failure mode is practically relevant: nine of the ten exhibit positive width bias. This motivates evaluating width directly at ROIs and reporting a within-tolerance ROI rate rather than inferring measurement quality from pixel overlap.
2. **We demonstrate that the bias can be corrected in post-processing, without retraining.** We use PCF as a lightweight correction layer that intersects a segmentation mask with a classical contrast-enhanced mask to remove systematic over-segmentation while preserving crack continuity. It attaches to any segmentation algorithm without retraining, given an operating point that over-segments. We show that PCF collapses nine of ten architectures into a consistent width accuracy. The one exception is an algorithm that under-segments, for which the correction has nothing to trim and width accuracy is unimproved.
3. **We perform measurement-based validation across two width-labeled datasets.** We evaluate ten algorithms on an uncontrolled field set (15 cracks, 75 physical ROIs yielding 225 caliper-referenced image–ROI observations) and an external set of 19,098 operator-measured width ROIs drawn from 36 image stacks of a single concrete material, report the within-tolerance ROI rate at AASHTO tolerances with image-clustered confidence intervals, and provide paired inferential tests (Wilcoxon and McNemar) for absolute error, coverage, and tolerance compliance. This contrasts with the IoU/Dice validation typical of the literature.

By shifting the objective from pixel-wise overlap to inspector-grade width measurement, PCF aligns machine vision with the decisions inspectors actually make. It integrates into existing workflows, improving the reliability and consistency of crack-width reporting without retraining upstream models or replacing the human inspector.

## 2. Related Work

### 2.1. Crack Segmentation: From Image Processing to Modern Algorithms

Crack analysis in images spans three tasks: detection (is a crack present, and where), segmentation (pixel-level delineation), and quantification (physical measures such as width, length, area, or density) [8,9]. Image-level classifiers and object detectors find cracks and their approximate location effectively [10,11,12,13,14], but width measurement requires pixel-precise segmentation. Early work relied on digital image processing (DIP)—thresholding, edge detection, morphology, region filtering, and skeletonization [15,16,17,18]. DIP methods are light and interpretable and can perform well in high-contrast scenes, but they are sensitive to lighting, texture, and shadows, need careful parameter tuning, and can fragment thin cracks [17]. Convolutional networks then became standard for pixel-level delineation: FCN and U-Net families [6,19,20,21], variants with residual, attention, or multi-scale modules [7,22,23,24], and hierarchical side-output designs such as DeepCrack [25], with reported IoUs of roughly 0.7–0.9 on curated datasets [6,11,21,23,25]. Still, thin, low-contrast, or textured cracks are missed or over-segmented, and performance drops in field conditions [8,14,23,26,27,28]. Newer families extend this line: crack-specific high-resolution networks (HRSeg) [29], CNN–transformer hybrids targeting fine topology and cross-material generalization (Hybrid-Segmentor) [30], foundation-model adaptations of Segment Anything [31,32], efficient state-space (Mamba) models (SCSegamba) [33], and robustness-oriented systems for low-light and zero-shot capture [34,35]. Practical systems also combine learned segmentation algorithms with classical refinement—connected components, filtering, distance transforms, skeletonization—or integrate UAV capture and calibration with advanced algorithms [14,26,36,37,38,39,40]. In these hybrids, however, the post-processing is usually a downstream cleanup of a single ML output, without an explicit cross-evidence agreement step. Our study spans these families deliberately—lightweight CNN, high-resolution CNN, transformer, Mamba, hybrid, and few-shot/low-light—so the conclusions are not tied to one segmentation algorithm.

### 2.2. From Masks to Measurements

The measurement literature repeatedly finds that segmentation and width estimation are distinct problems: width depends on centerline stability, boundary fidelity, local normal direction, branching logic, and physical scale, so systems that actually report widths retain an explicit geometric stage even when the segmentation algorithm is learned [14,39]. Representative estimators use skeleton or medial-axis extraction, principal-axis estimation, optimal-path tracking, and boundary-aware orthogonal-projection sampling [41,42]; multi-task designs treat quantification as a first-class output [43]. PCF is complementary to all of these: every geometric width estimator consumes a mask, and PCF improves the mask that any such estimator receives. Evaluation, however, remains dominated by overlap metrics—precision, recall, F1, IoU, pixel accuracy [10,12,23,25]—and overlap does not certify physical width accuracy [9,44]: as quantified in the Introduction, an over-segmented strip mask inflates measured width by approximately 1/IoU, consistent with prior reports of more than ±25% width error despite high IoU [9]. We therefore treat overlap as a secondary diagnostic and lead with measurement-first quantities: absolute width error in millimeters, the within-tolerance ROI rate, and the rate of missing measurements, all evaluated against operator ground truth [14,26].

### 2.3. Benchmarks, Gap, and Positioning

Segmentation datasets have grown far faster than width-labeled measurement benchmarks: most recent corpora emphasize classification, detection, or segmentation rather than metric width, so a crack-width paper needs a second, width-labeled evaluation. We complement our field set with an external, operator-measured dataset providing thousands of width-labeled ROIs, so the central claim is validated on physical width rather than mask overlap alone. Across DIP-only, deep, and hybrid pipelines, two gaps persist. First, few systems enforce consensus between independent evidence streams to correct recall-driven boundary inflation without breaking continuity; most enhance images before inference or clean a single ML mask afterward. Second, evaluation remains overlap-centric, with limited emphasis on inspector-facing outcomes: millimeter-accurate width, measurement completeness, and scale consistency. PCF targets both gaps: a measurement-reliability layer, applicable to any segmentation algorithm whose operating point over-segments, that corrects a measurable, systematic over-segmentation bias through cross-evidence consensus, evaluated on physical width across ten algorithms and two datasets.

## 3. PCF Algorithm Methodology

The PCF algorithm is not intended as a contribution by itself, but rather as a way to test the bias correction hypothesis. It refines the mask from any segmentation algorithm into accurate, resolution-robust crack-width measurements in millimeters by fusing it with a complementary classical image-processing stream, rather than training a new network.

**Figure 1:**
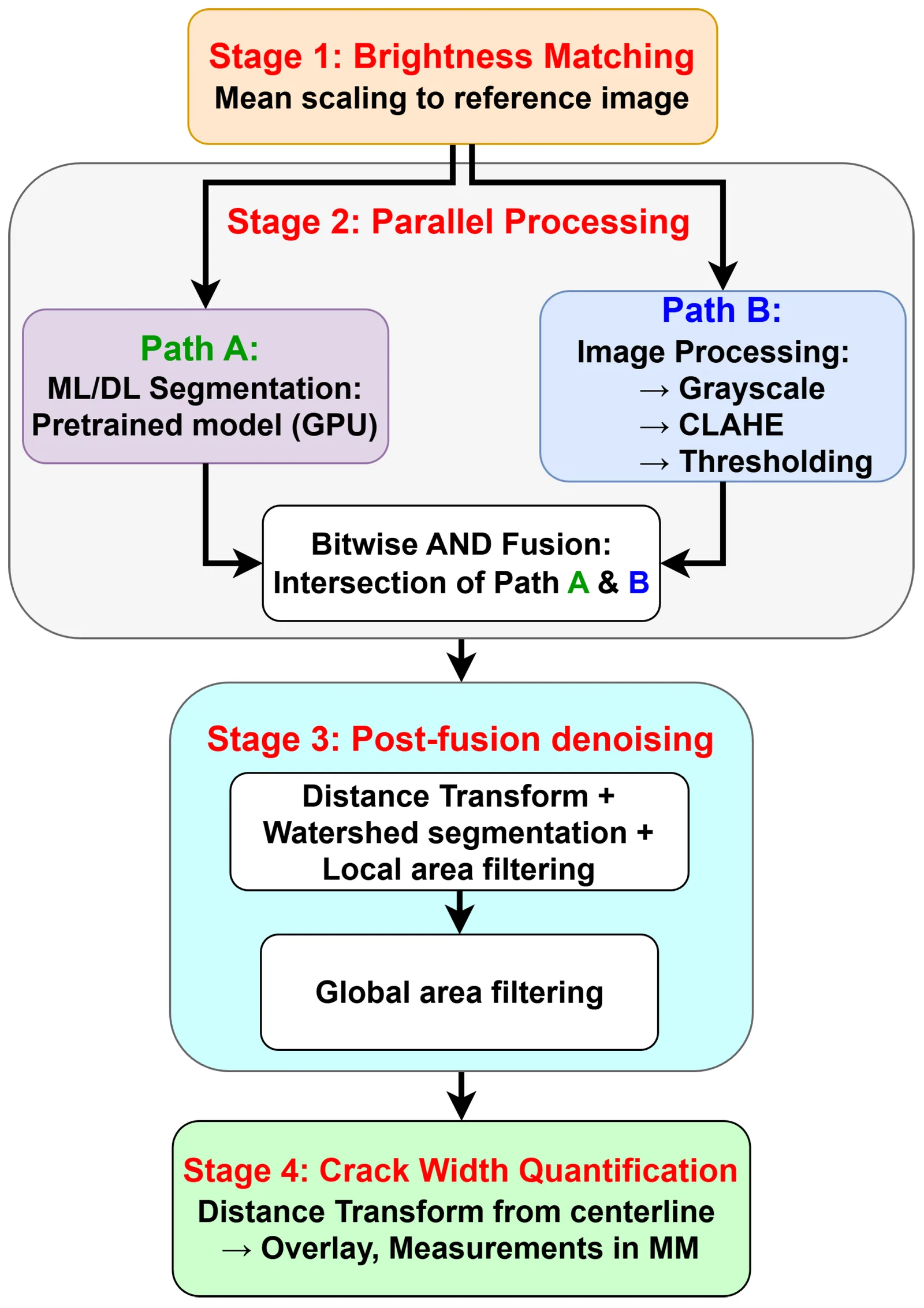
Parallel Consensus Fusion pipeline: brightness matching, ML segmentation (Path A), CLAHE-and-threshold segmentation (Path B), consensus fusion, topology-aware denoising, and distance-transform-based width extraction.

As shown in Figure 1, the pipeline uses a parallel consensus-fusion layout. Path A produces a continuity-rich mask from the ML segmentation model, while Path B produces an edge-sharp mask via contrast enhancement and thresholding. Both operate independently on the same brightness-matched image and are merged by a pixelwise logical AND: the intersection trims the directional over-segmentation that inflates measured width while Path A preserves crack extent, so the correction helps most where the segmentation algorithm over-segments and is unhelpful where it under-segments. Minimal fixed-parameter denoising then removes speckle and shallow attachments, and calibrated widths are obtained from a two-sided distance transform. Throughout, we target inspector outcomes: millimeter accuracy, measurement completeness across ROIs, and stability under resolution and intra-crack width variation.

### 3.1. Brightness Matching (Stage 1)

Given an input RGB image *I* and a reference field image $I_{\mathrm{ref}}$, we match global mean intensity using a scalar gain. Let μ(·) denote the mean pixel intensity averaged over all spatial locations and color channels, and let ε be a small constant to avoid division by zero. The gain *k* is

(1) $$k=\frac{\mu \;\left(I_{\mathrm{ref}}\right)}{\mu (I)+\varepsilon }\;,$$

and the brightness-matched image is

(2) $$I^{\prime }=\mathrm{clip}\left(kI\right)\;,$$

where clip(·) clamps values to [0,255]. The same $I^{\prime }$ is provided to both paths, with no other coupling between them. $I_{\mathrm{ref}}$ is a single, fixed reference image from the field campaign, chosen once for typical exposure; because the matching is only a scalar gain on the global mean and the downstream Path B threshold is calibrated per domain (Section 3.5), moderate changes in the choice of $I_{\mathrm{ref}}$ are absorbed by that calibration rather than propagating to the measurements.

### 3.2. Parallel Processing (Stage 2)

In Path A, the chosen algorithm processes $I^{\prime }$ and produces a probability map $P_{A}\left(x\right)$ over pixel locations *x*. A fixed threshold θ yields a binary mask

(3) $$A\left(x\right)=I\;\left[P_{A}\left(x\right)\geq \theta \right]\;,$$

where I[·] is the indicator function. In field imagery, recall-favoring thresholds typically preserve continuity but can over-segment boundaries.

We evaluate PCF across *ten* segmentation algorithms—the learned models that produce A(x), commonly referred to as segmentation backbones—chosen to span architectures and capacities (roughly 1.2–238 M parameters), so that the conclusions cannot be attributed to a single algorithm. Six are established crack-segmentation networks—RUCNet [45], DeepCrack [25], HRSeg [29], U2CrackNet [23], UNet-FocalLoss [21], and DeepLabv3+ [24,46]—and four are recent architectures spanning current design trends: the vision-Mamba model SCSegamba [33], the transformer CrackFormer-II [47], the few-shot/low-light model CrackNex [34], and the CNN–transformer hybrid Hybrid-Segmentor [30]. All models except DeepLabv3+ use publicly available pretrained weights. For DeepLabv3+, we trained an in-house model at 512×512 input resolution on a composite corpus that includes Crack500 [44], DeepCrack [25], CrackTree [17], GAPs [48], Rissbilder [49], CFD [50], Sylvie Chambon [51], and Volker [49].

Each algorithm is run at the inference settings recommended by its authors or released project code; we do not tune the segmentation algorithms. Most of the algorithms produce binary masks directly (the PaddleSeg-integrated networks expose no probability threshold at all), so A(x) is taken as their output. Only DeepLabv3+ and DeepCrack expose a confidence threshold in our harness; on the field set these are fixed at reasonable recall-favoring values (DeepLabv3+: θ=0.85; DeepCrack: θ=0.40)—chosen once, before the evaluation, so that few cracks are missed outright—and the consensus fusion step then trims the over-segmented boundaries this produces. We make no claim that these are optimal thresholds; the point is that they sit in the recall-favoring regime practitioners typically use. This asymmetry is deliberate: PCF is the calibrated layer under test, while the segmentation algorithms are used as practitioners receive them. The over-segmentation PCF corrects originates upstream of any other threshold—in training-label thickness and recall-weighted losses—so it is a property of the mask family rather than of a particular θ; what θ does control is the *sign* of the bias, which is exactly the regime behavior examined in the Results (Section 3.5, Table 1).

In Path B, we apply classical image processing to $I^{\prime }$. The image is converted to grayscale and enhanced using contrast-limited adaptive histogram equalization (CLAHE), then binarized with a global grayscale threshold to produce a binary mask B(x). The CLAHE tile and clip and the grayscale threshold are calibrated operating points rather than universal constants. The values used in each domain are listed in Section 3.5, Table 1. This path yields sharp edges but is more prone to small holes and speckle on textured, stained, or low-contrast surfaces.

The two masks are fused by a pixelwise logical AND,

(4) F(x)=A(x)∧B(x),

so a pixel is retained only when both paths label it as crack. The consensus mask *F* typically preserves the continuity of Path A while tightening boundaries using Path B: it trims over-segmented margins, reduces isolated false positives, and preserves a connected centerline wherever both streams detect the crack.

**Figure 2:**
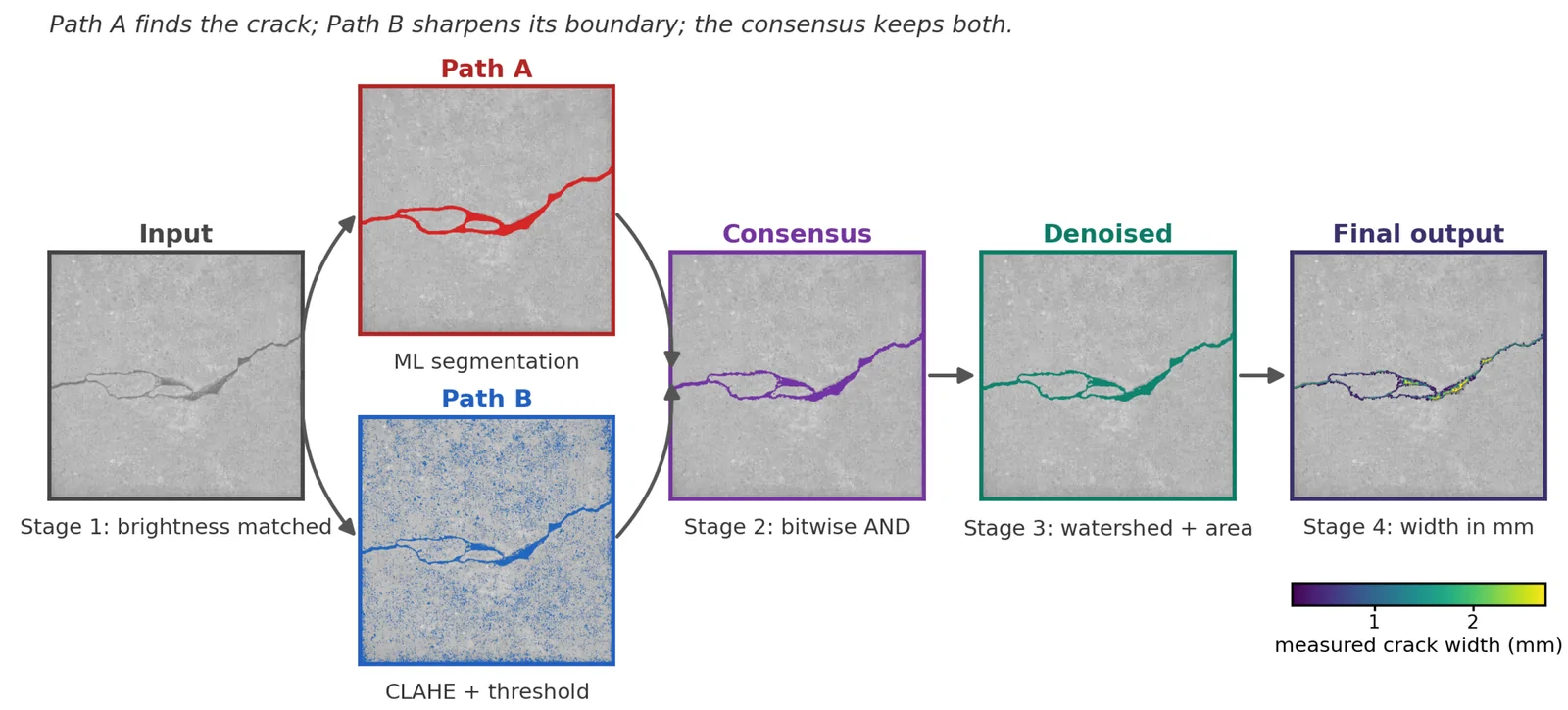
A single field image carried through the PCF pipeline (Sample 2 at 0.5 m; SCSegamba as Path A). Path A segments the crack but over-segments its width, while Path B provides sharper boundaries but cannot independently identify the crack. Their pixelwise intersection retains Path A’s localization with Path B’s tighter boundary. Stage 3 removes residual speckle, and Stage 4 extracts local width along the medial axis. Masks are overlaid on the grayscale image; the two thinnest masks are slightly dilated for print legibility.

The division of labor between the two paths is worth stating precisely, because it governs how the ablation in Section 5.4 should be read. **Path B does not segment the crack.** A global threshold on CLAHE-enhanced imagery fires on stains, texture, shadows, and aggregate boundaries everywhere in the frame (Figure 2), so it cannot distinguish crack pixels from any other dark pixels; what it does well is place a sharp boundary once it is told where to look. Path A does the segmentation—it is the only stage that identifies which part of the image is the crack—and Path B then sharpens the boundary of what Path A found. The consensus inherits both properties. This matters for evaluation: when Path B alone is scored at ROIs whose locations the dataset supplies, it is being handed the answer to the part of the problem it cannot solve, so its stand-alone accuracy is an upper bound on reading a width at a known location, not evidence that it could work alone in deployment.

### 3.3. Post-Fusion Denoising (Stage 3)

The fused mask *F* may still contain speckle and small blobs, so we apply a watershed on a smoothed distance map followed by two area filters. All constants below are fixed across the dataset; none is tuned per image or per ROI.

We compute the Euclidean distance transform inside *F*, smooth it with a 15×15 Gaussian kernel (σ=3 px) to obtain a topographic surface T(x), normalize *T* to [0,1], and apply an *h*-minima transform with $h_{\min}=0.02$. Thick crack interiors and blobs appear as peaks, while gaps between fragments appear as valleys; the *h*-minima step fills valleys shallower than $h_{\min}$, which closes gaps shorter than the smoothing kernel while leaving wider gaps as distinct valleys. A watershed on −T(x) then partitions the foreground into basins $R_{k}$, for each of which we take the pixel area $A_{\mathrm{local}}\left(k\right)$ and the bounding-box aspect ratio

(5) $$AR_{\left(R_{k}\right)}=\frac{\min(w_{k},h_{k})}{\max(w_{k},h_{k})}\;,$$

where $w_{k}$ and $h_{k}$ are the box width and height. Because cracks are long and thin, basins with $AR_{\left(R_{k}\right)}\leq 0.25$ are treated as crack-like and define a local corridor; rounder basins are treated as non-crack features. Two filters follow. Within the corridor, basins smaller than $A_{\min}^{\mathrm{local}}=25$ px are suppressed, removing speckle and tiny side branches while leaving the crack intact. Then a global 8-connected component filter discards components smaller than $A_{\min}^{\mathrm{global}}=70$ px, clearing isolated blobs outside the corridor. Both thresholds were selected once on a small validation set. The result is the final mask $M^{⋆}$ passed to crack-width quantification.

### 3.4. Crack-Width Quantification (Stage 4)

We compute a distance transform on $M^{⋆}$ and extract its ridges, which approximate the medial axis of each crack. At each medial-axis pixel *p*, we follow the local normal direction to the left and right until the crack boundary to obtain one-sided distances $d_{L}\left(p\right)$ and $d_{R}\left(p\right)$ in pixels. The local width in pixels is $w_{\mathrm{pix}}\left(p\right)=d_{L}\left(p\right)+d_{R}\left(p\right)$. Using the per-image calibration factor *s* in pixels per millimeter, obtained from the 150 mm reference square, the physical width is $w_{\mathrm{mm}}\left(p\right)=w_{\mathrm{pix}}\left(p\right)/s$. Because the measurement follows the local normal and does not assume symmetry, it remains valid for tapered or irregular edges. The choice of a distance-transform medial axis with normal-direction sampling is empirical rather than assumed: we benchmarked 38 width-estimator variants spanning nine method families (skeleton, contour, Feret, normal-ray, and consensus variants, among others), and the adopted method attained the lowest width error.

### 3.5. Calibration of Operating Points

PCF exposes only a small number of parameters: the Path B grayscale threshold, the CLAHE clip limit and tile size, and two post-fusion filters (a minimum cluster size and a topology threshold). These parameters are calibrated on the internal field dataset and then fixed. Calibration is coverage-constrained: over a grid of grayscale-threshold and CLAHE values, we select the operating point that maximizes the within-tolerance ROI rate while maintaining at least a target ROI coverage. The selected setting is then applied uniformly to all images and ROIs; no per-image or per-ROI tuning is performed. The coverage constraint prevents trivial reductions in error obtained by discarding difficult ROIs. Width error varies smoothly across the grid and the chosen point lies on the error–coverage frontier, so the method is not balanced on a knife’s edge.

For the six established CNN algorithms, the field-set configuration (CLAHE clip 2.0, 15×15 tiles, grayscale threshold 15) was fixed in our earlier field study and is carried over unchanged. For the four modern RGB algorithms, a single shared configuration (CLAHE clip 4.0, 8×8 tiles, grayscale threshold 30, intersection fusion) is used across the family. A leave-one-crack-out calibration check (Appendix A.3) selects the same operating point in every fold, indicating that this configuration is not driven by any individual crack or ROI.

External evaluation on krkCMd is performed without calibration to the external width references. The PCF settings selected on the internal field data are frozen before external testing and transferred to krkCMd without using krkCMd ground-truth widths for parameter selection. Dataset-specific changes in image preprocessing or segmentation-model inference settings, where required by the released implementations, are treated separately from PCF calibration and are reported explicitly. The krkCMd results therefore evaluate cross-domain transfer of an internally calibrated PCF procedure rather than on-domain tuning.

Table 1 records every parameter the pipeline exposes, the data it was selected on, and the reference consulted; no krkCMd width reference was used for parameter selection. Across the grid (grayscale threshold 5–30, CLAHE clip 1.0–4.0) the within-tolerance ROI rate varies smoothly and monotonically, and the selected point is the grid optimum for every algorithm in the family, lies on the error–coverage frontier, and is re-selected unchanged under every coverage floor from 0% to the maximum attainable (Appendix E); it also sits at the grid’s upper bound on both axes, so a better setting may exist outside it. One selection warrants mention: the Stage 4 estimator was chosen on the same 225 internal ROIs later used for evaluation. It is identical for every algorithm and both arms, so it cannot create a differential advantage, and the paired comparisons are unaffected.

## 4. Experiment Design

### 4.1. Study Framing and Comparison Logic

The experiments are designed to test three aspects of PCF: whether segmentation error is systematically directional, whether consensus fusion reduces that error without retraining, and whether the resulting improvement is attributable to the fusion stage and remains robust across test conditions. These questions are formalized as hypotheses in Section 4.7.

For each algorithm, the primary comparison is paired within ROI: the ML-only control passes the Path A mask directly to width quantification, whereas PCF fuses Path A and Path B before quantification. Stage 4 is held fixed, so differences between the two configurations arise from the mask. A four-way external ablation (Section 5.4) additionally compares ML-only, ML + denoising, Path B only, and consensus fusion to identify the stage responsible for the observed gains.

### 4.2. Field Data Acquisition and Setup

**Figure 3:**
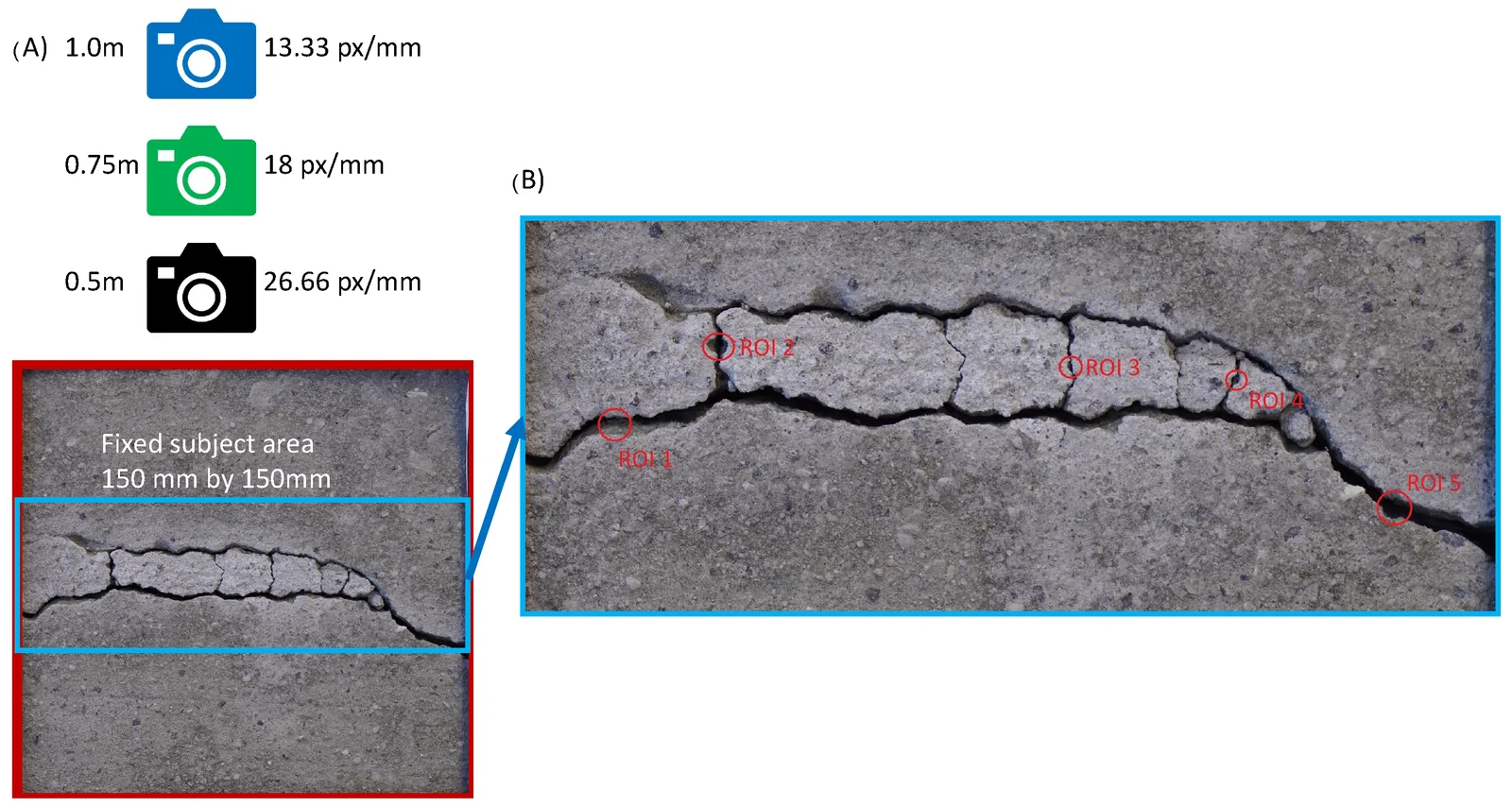
(**A**) Capture distances and corresponding effective resolutions for the crack images. (**B**) Example crack image with five annotated ROIs used for width-quantification benchmarking.

To approximate real-world field inspection conditions, we collected 45 images of concrete cracks using a Sony A6000 mirrorless camera with a fixed 55 mm lens. Each image included a printed 150mm×150mm calibration square for estimating image resolution. Fifteen distinct cracks were imaged once at standoff distances of 1.0 m, 0.75 m, and 0.5 m (Figure 3), corresponding to effective resolutions of approximately 13.33, 18.00, and 26.66 pixels per millimeter, respectively. This repeated-distance design enables direct evaluation of resolution robustness on the same physical cracks.

The cracks were located on building façades, bridge components, and concrete floor slabs around the Virginia Tech campus. Imaging was intentionally uncontrolled: photographs were taken outdoors at different times of day (sunrise, midday, and sunset) and under sunny and overcast conditions. Surfaces were not cleaned, and shadows, dirt, efflorescence, and moisture were retained. No tripod was used, preserving natural hand motion and variability representative of routine inspection.

### 4.3. Region of Interest Selection and Ground Truth Collection

For each of the 15 cracks, five regions of interest (ROIs) were manually selected once, before automated processing, by a single trained operator. ROIs were distributed along each crack to provide spatial coverage and variation in thickness, orientation, background texture, and lighting. Several cracks also contained branching, web-like, or tapered regions, providing within-crack width variation in addition to the resolution changes introduced by capture distance. An example is shown in Figure 3.

At each ROI, crack width was measured with digital calipers (resolution 0.03 mm/$0.001^{\prime \prime }$, repeatability 0.01 mm/$0.0005^{\prime \prime }$) in three trials and averaged to obtain the reference width. Pooling the per-ROI variances over the 75 physical ROIs gives a single-operator repeatability standard deviation of 0.057 mm, so the three-trial mean carries an expanded uncertainty of ±0.064 mm. Repeatability scales with width, from ±0.030 mm below 2 mm to ±0.119 mm above 4 mm, so for 8 of the 75 ROIs, all wide cracks, the reference uncertainty alone exceeds the tolerance band. These figures are single-operator repeatability and do not establish inter-observer reliability. Because AASHTO condition states use absolute width bands, we use ±0.10 mm as the primary evaluation tolerance, matching both the reference repeatability at these small widths and the AASHTO measurement tolerance [1]. The same physical measurements were associated with all three capture distances using the calibration square.

Across the 75 physical ROIs (15 cracks × 5 ROIs), approximately 13% had widths ≤0.1 mm and another 22% were below 0.5 mm. At least six cracks contained these narrow features, providing cases near the resolution limit.

The 45 images contain repeated observations of the same 75 physical ROIs at three capture distances, yielding 225 image–ROI observations. The ROI therefore serves as the measurement unit for accuracy analyses, with capture distance treated as a repeated imaging condition.

### 4.4. Primary Evaluation Metrics

Because crack width, rather than mask overlap, drives inspection decisions, we evaluate all methods on the same fixed set of ROIs with operator width references. At each ROI, the pipeline may return a width, and that width may or may not fall within the tolerance band τ.

- **Absolute width error (mm)**—|measured−reference| at each ROI where a width was returned. Reported as a distribution; its mean is the MAE.
- **Coverage (%)**—the percentage of ROIs at which the pipeline returns *any* width. Coverage is lost when the mask is empty or broken at the ROI, so the crack cannot be measured there. Coverage says nothing about whether the returned width is correct.
- **Within-tolerance ROI rate (%)**—the percentage of all ROIs at which a width is both returned and within τ of the reference. Equivalently, coverage multiplied by the share of returned widths that are correct. This is the paper’s primary outcome, because an ROI that is covered by a badly biased width is not usable to an inspector, and an ROI with no width at all is not usable either; only this metric penalizes both failures.

The distinction is substantial in practice: on krkCMd, DeepLabv3+ returns a width at 90.0% of ROIs but only 2.4% are within tolerance. We evaluate the within-tolerance rate primarily at ±0.10 mm, with the AASHTO ±0.20 and ±0.30 mm allowable-error bands as robustness checks [1].

IoU and Dice are not used as success metrics because, as established in the Introduction, high overlap does not certify width accuracy. They also cannot be computed on krkCMd, which provides operator width references along grid lines but no ground-truth crack masks. Coverage therefore serves as the available indicator of whether a measurable crack mask is present at the reference ROI. This also precludes a direct numerical comparison with published width-quantification methods: they are evaluated on different datasets against different references, and most report overlap metrics or relative error rather than metric width against a physical reference. Reported numbers are therefore not commensurable, which is itself the gap this work addresses.

### 4.5. Physical Scale and Capture Distance

For each image, the millimeters-per-pixel scale is computed from the calibration square as $k=150\;\mathrm{mm}/N_{\mathrm{edge}}$, where $N_{\mathrm{edge}}$ is the pixel length of one cropped square edge. Equivalently, the pixels-per-millimeter factor used in Stage 4 is s=1/k. Measured widths are converted using $w_{\mathrm{mm}}=k\;w_{\mathrm{px}}$. Placing the square on the cracked surface reduces depth mismatch, and measuring its apparent size avoids reliance on the nominal capture distance. However, a single scalar calibration does not fully correct orientation-dependent foreshortening, spatially varying perspective, lens distortion, or surface non-planarity. Across the 45 images, scale inferred from nominal distance differed from the square-based scale by a median of 1.4% and by up to 10.0%. The internal analyses pool the 225 image–ROI observations, with capture distance evaluated separately as a repeated robustness factor in H5 (Section 5.5).

### 4.6. External Validation Dataset

For external validation, we use the public krkCMd dataset [52], comprising high-resolution scans of self-healing concrete with 19,098 grid-line ROIs across six progressive healing stages. Each ROI has an operator ImageJ2 (version 2.9.0/1.53t) width reference. Crack widths are sub-millimeter (median ≈0.12 mm), complementing the larger cracks in our field set. The native scans are 9448×6305 px (3.97μm/px), so even sub-millimeter cracks span tens of pixels; reference uncertainty is characterized in the dataset’s technical validation [52].

The evaluation follows the internal protocol: all ten algorithms are evaluated before and after fusion using the same measurement-based metrics. Frames are processed whole, without tiling or cropping. For inference, probability-map models are resized to a 2000 px long side and the modern algorithms to 0.25× area; predicted masks are then mapped back to the measurement resolution. Width is read at the reference measurement location: at the caliper probe point internally and, on krkCMd, along the supplied grid line as the contiguous crack-pixel run nearest the brightness-profile minimum.

krkCMd also provides two profile-based width estimates, DLMwidth and AEDwidth. Both measure along supplied grid lines and perform no crack detection or segmentation. We therefore report them as dataset-native reference points for width measurement at a known location, not as head-to-head comparators for the segmentation-then-measurement pipeline. The Path B-only ablation (Section 5.4) provides the analogous controlled comparison within our pipeline.

A quarter of the external references (25.7%) are exactly 0.000 mm, corresponding to fully healed grid lines; the median over non-zero references is 0.175 mm. The 19,098 ROIs come from only 36 physical image stacks of a single material and are therefore not treated as independent. External confidence intervals use a cluster bootstrap that resamples the 36 stacks.

### 4.7. Hypotheses

The analysis is organized around five hypotheses, stated in the order in which the Results test them. Each has a dedicated Results subsection, indicated in parentheses, so the reader can follow one claim at a time on the same per-ROI data.

**Hypothesis** **1** **(H1).** ***The error is a directional bias.** Crack-width error from a segmentation algorithm alone is dominated by systematic over-segmentation—measured widths are too large—rather than by random scatter (Section 5.1).*

**Hypothesis** **2** **(H2).** ***The bias is correctable in post-processing.** Consensus fusion reduces the per-ROI absolute width error relative to the ML-only control, without retraining the segmentation algorithm (Section 5.2).*

**Hypothesis** **3** **(H3).** ***The correction produces usable measurements.** PCF raises the within-tolerance ROI rate at the ±0.10 mm band, and it does so by more than it costs in coverage (Section 5.3).*

**Hypothesis** **4** **(H4).** ***Consensus fusion is the operative stage.** The gain comes from the intersection of the two paths specifically—not from denoising the ML mask, and not from Path B, which cannot segment the crack on its own (Section 5.4).*

**Hypothesis** **5** **(H5).** ***The correction is robust.** The improvement holds across capture distance, across the looser AASHTO tolerance bands, and across crack condition from open to nearly healed (Section 5.5).*

## 5. Results

Results are presented in the order of hypotheses H1–H5 from Section 4.7, followed by the formal paired statistical tests.

Unless noted, internal results use N=225 image–ROI observations across three capture distances, while external results use all 19,098 krkCMd ROIs with cluster-bootstrap confidence intervals over the 36 image stacks. Hypothesis tests use the strict ±0.10 mm tolerance; the looser AASHTO bands are examined in Section 5.5. Absolute-error outliers (Tukey 1.5 × IQR) are retained, and sensitivity checks did not change the conclusions.

The two datasets differ substantially in physical image scale. A 0.10 mm tolerance corresponds to approximately 1.3, 1.8, and 2.7 px at the 1.0, 0.75, and 0.5 m field distances, respectively, but about 25 px on native krkCMd scans (3.97μm/px). Absolute within-tolerance rates should therefore not be compared directly across datasets; the relevant cross-dataset comparison is the direction and consistency of the PCF effect.

### 5.1. H1: The Width Error Is a Directional Over-Segmentation Bias

**Figure 4:**
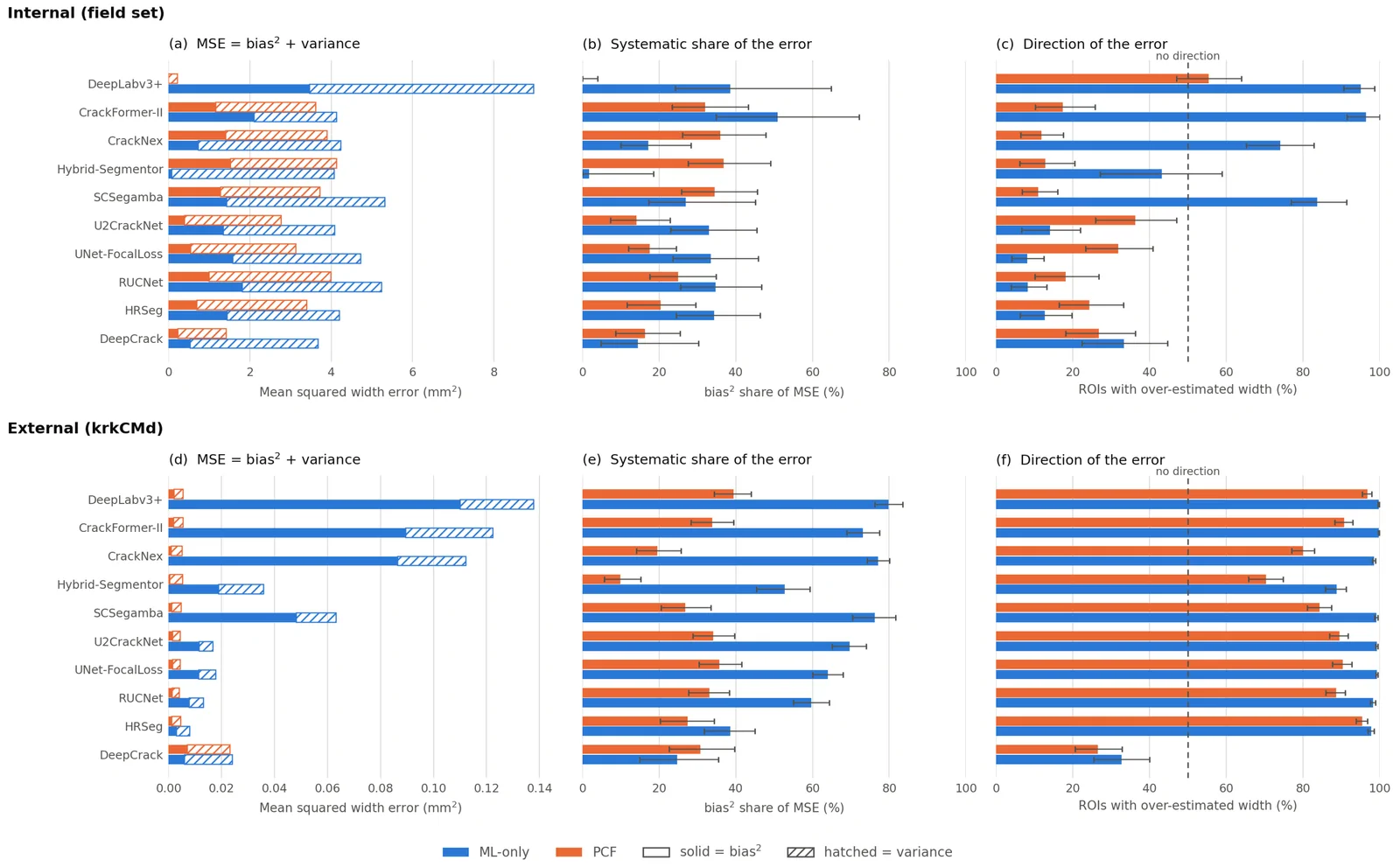
Error structure on the internal field set (top) and krkCMd set (bottom). (**a**,**d**) Mean squared width error divided into ${\mathrm{bias}}^{2}$ (solid) and variance (hatched); the two rows use different scales. (**b**,**e**) Percentage of MSE explained by ${\mathrm{bias}}^{2}$. (**c**,**f**) Percentage of ROIs with over-estimated widths. This measure uses only the sign of each error. The dashed line at 50% represents equal rates of over- and under-estimation. Error bars show 95% cluster-bootstrap confidence intervals, with resampling over the 15 field cracks and 36 krkCMd image stacks.

Signed errors on the krkCMd set are mostly positive, which indicates width over-estimation (Figure 4d–f). Nine of the ten algorithms show this pattern. DeepLabv3+ and CrackFormer-II have the largest mean signed errors, at +0.33 and +0.30 mm, respectively. Their estimated widths average 3.0 and 2.7 times the reference width. This result is consistent with the geometric over-segmentation mechanism described in the Introduction.

A positive mean could be caused by a small number of large errors. We therefore use two additional measures to determine whether the bias is systematic: the fraction of mean squared error (MSE) explained by ${\mathrm{bias}}^{2}$, and the percentage of ROIs with positive error. The second measure depends only on the direction of each error and is not affected by its magnitude. Across the nine over-estimating algorithms, ${\mathrm{bias}}^{2}$ accounts for 38.6–79.9% of the external MSE, and 88.7–99.8% of measurable ROIs are over-estimated. DeepLabv3+, for example, over-estimates 99.7% of its 17,187 measurable ROIs.

After fusion, the errors become less directional. For DeepLabv3+, the fraction of MSE explained by ${\mathrm{bias}}^{2}$ decreases from 79.9% to 39.4%. This indicates that fusion removes much of the systematic bias rather than simply reducing all errors by the same amount. Fusion also reduces MSE for nine of the ten algorithms on the field set. The largest reduction occurs for DeepLabv3+, whose MSE decreases from 8.99 to 0.22 mm^2^.

Bias direction is not determined by architecture alone. It also changes with crack width and the selected operating point. The field cracks are much wider than the external cracks, with median reference widths of 1.75 and 0.119 mm, respectively. Only four of the ten algorithms over-estimate width on the field set. For example, HRSeg has a mean error of +0.108 mm for cracks narrower than 0.6 mm, but a mean error of −2.150 mm for cracks wider than 2 mm.

DeepCrack is the only algorithm that under-segments on the external set at its selected operating point. Because fusion uses an intersection, it cannot recover crack pixels that are already missing from the upstream mask. Fusion is therefore expected to help the nine over-estimating algorithms, but not DeepCrack, as evaluated in Section 5.2, Section 5.3 and Section 5.4.

### 5.2. H2: Consensus Fusion Removes the Bias and Lowers the Width Error

Figure 4a,d shows that fusion reduces the signed bias. We next evaluate whether it also reduces total error using the absolute width error at each ROI.

**Figure 5:**
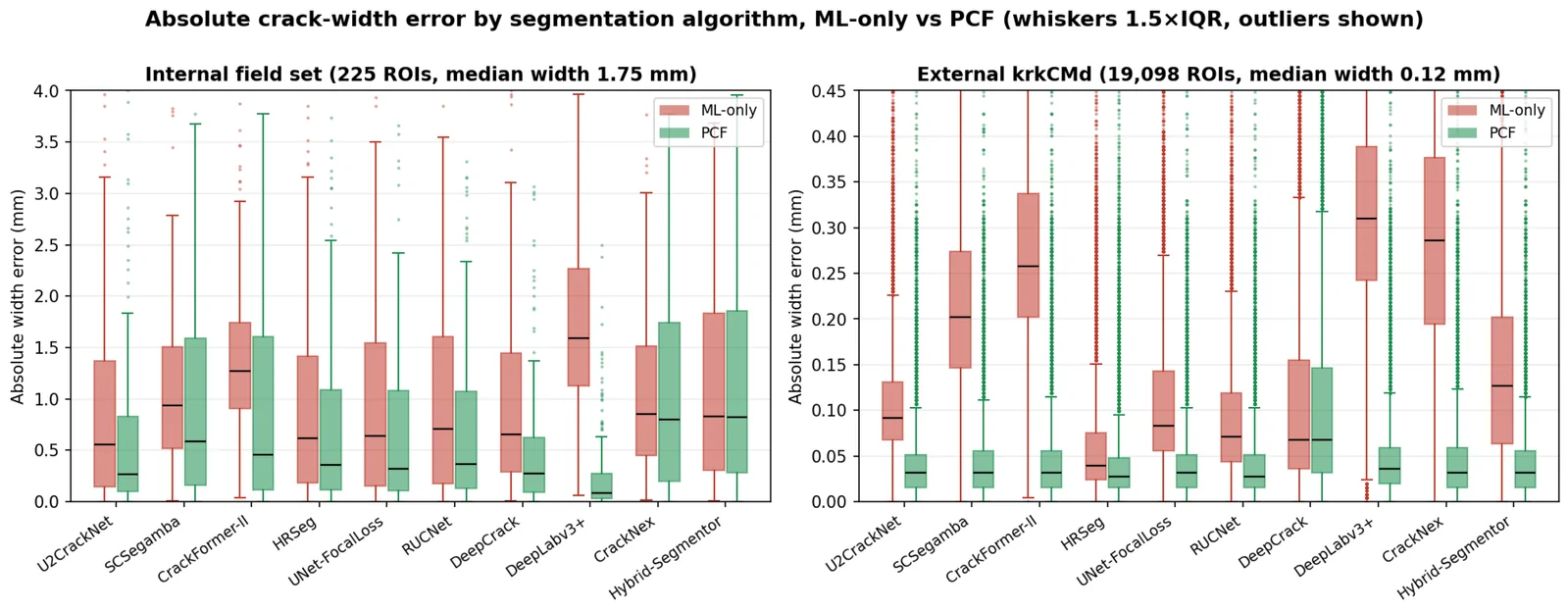
Absolute crack-width error per ROI, ML-only vs. PCF, on the internal field set (**left**) and the external krkCMd set (**right**). Boxes show the interquartile range and median; whiskers extend to 1.5 × IQR and individual outliers are plotted (axes clipped at 4.0 and 0.45 mm for readability). PCF lowers and tightens the error distribution for nearly every algorithm; DeepCrack on krkCMd is the documented exception.

Figure 5 shows that PCF lowers and tightens the error distribution for nearly every algorithm on both datasets. The largest reductions occur for the strongest over-segmenters: on krkCMd, DeepLabv3+ falls from 0.333 to 0.051 mm mean absolute error and CrackFormer-II from 0.300 to 0.048 mm, while HRSeg changes only from 0.062 to 0.044 mm. Before fusion, the ten algorithms span 0.062–0.333 mm in external mean absolute error; after fusion, nine cluster within 0.042–0.051 mm—a 9μm range—despite spanning 1.2–238 M parameters. DeepCrack, the under-segmenting exception identified in Section 5.1, changes little (0.111 to 0.107 mm).

**Figure 6:**
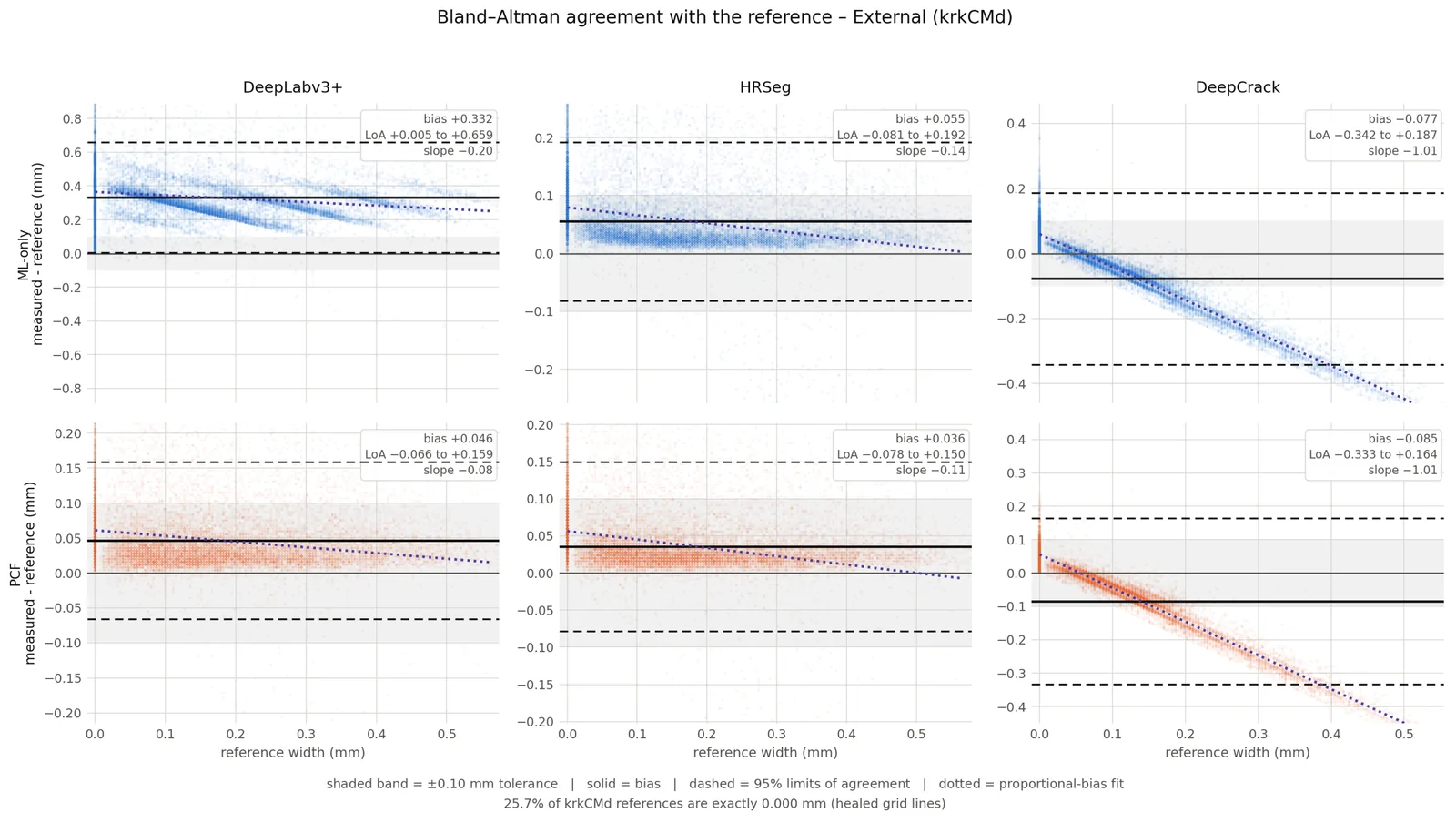
Bland–Altman agreement with the operator reference on krkCMd, before (**top**) and after (**bottom**) fusion, for a strong over-segmenter (DeepLabv3+), a near-unbiased algorithm (HRSeg), and the under-segmenting exception (DeepCrack). Solid line, mean difference; dashed lines, 95% limits of agreement; dotted line, proportional-bias fit; shaded band, the ±0.10 mm tolerance.

Expressed as agreement with the operator reference (Figure 6), the span of the 95% limits of agreement falls from 0.653 to 0.225 mm for DeepLabv3+, with the nine over-segmenting algorithms converging to 0.206–0.270 mm from a spread of 0.273–0.711 mm. The correction also makes the returned width track the reference rather than merely sit closer to it: the correlation between measured and reference width rises from +0.56 to +0.91 for DeepLabv3+, and the nine converge to +0.87–+0.92. HRSeg, already near-unbiased, changes little (0.273 to 0.228 mm). DeepCrack does not improve, and its returned width is uncorrelated with the reference (r=−0.05), a case examined in Section 6. Agreement for all ten algorithms on both datasets is given in Appendix C.

### 5.3. H3: The Corrected Measurements Are Usable at Inspection Tolerance

**Figure 7:**
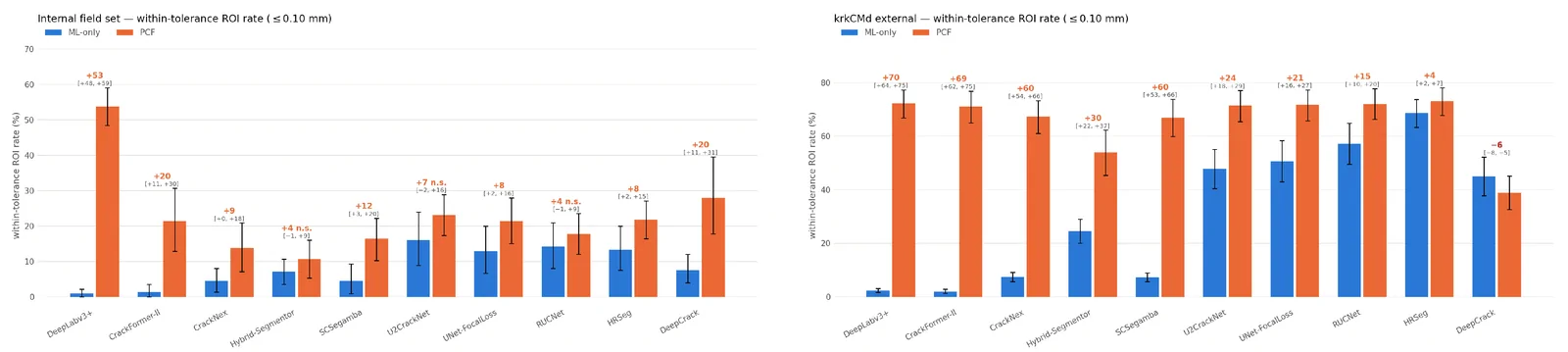
Within-tolerance ROI rate at ±0.10 mm for ML-only and PCF on the internal field set (**left**) and krkCMd (**right**). Labels show the change in percentage points. PCF improves 10/10 internal and 9/10 external algorithms; DeepCrack is the exception.

Figure 7 compares the within-tolerance ROI rate at ±0.10 mm before and after fusion. PCF increases the rate for all ten algorithms on the internal field set and for nine of ten on krkCMd; under crack-clustered resampling the internal gain excludes zero for seven of the ten (Appendix A.2).

The largest gains occur where baseline widths were least usable. On krkCMd, DeepLabv3+ rises from 2.4% to 72.2% within tolerance (+70 percentage points, pp), and CrackFormer-II from 2.0% to 71.1% (+69 pp). DeepLabv3+ coverage remains high, decreasing only from 90.0% to 81.5%, showing that its baseline failure was primarily inaccurate width estimation rather than failure to return a measurement. This illustrates why coverage alone does not indicate inspection usability.

PCF therefore trades some coverage for substantially higher measurement accuracy. This trade is favorable for nine of ten external algorithms. DeepCrack is the exception: coverage falls from 70.6% to 59.9% and the within-tolerance rate from 45.0% to 38.9% (−6 pp), consistent with its under-segmenting operating point identified in Section 5.1.

### 5.4. H4: Consensus Fusion, Not Denoising, Is the Operative Stage

To identify the stage responsible for the PCF gain, we ran a four-way ablation on all 19,098 external ROIs for all ten algorithms: ML-only, ML + denoising, Path B only, and PCF fusion. The ML-only and PCF arms use the same operating points reported elsewhere, and uncertainty is estimated with the same 36-stack cluster bootstrap.

The ML + denoising arm was calibrated separately because the Stage 3 parameters used by PCF were originally selected for post-fusion masks. Applying those settings directly to an unfused ML mask would therefore confound the ablation with an inappropriate operating point. The denoising-only arm was instead calibrated using the same coverage-constrained procedure, providing a fair test of whether denoising alone can reproduce the PCF gain.

**Figure 8:**
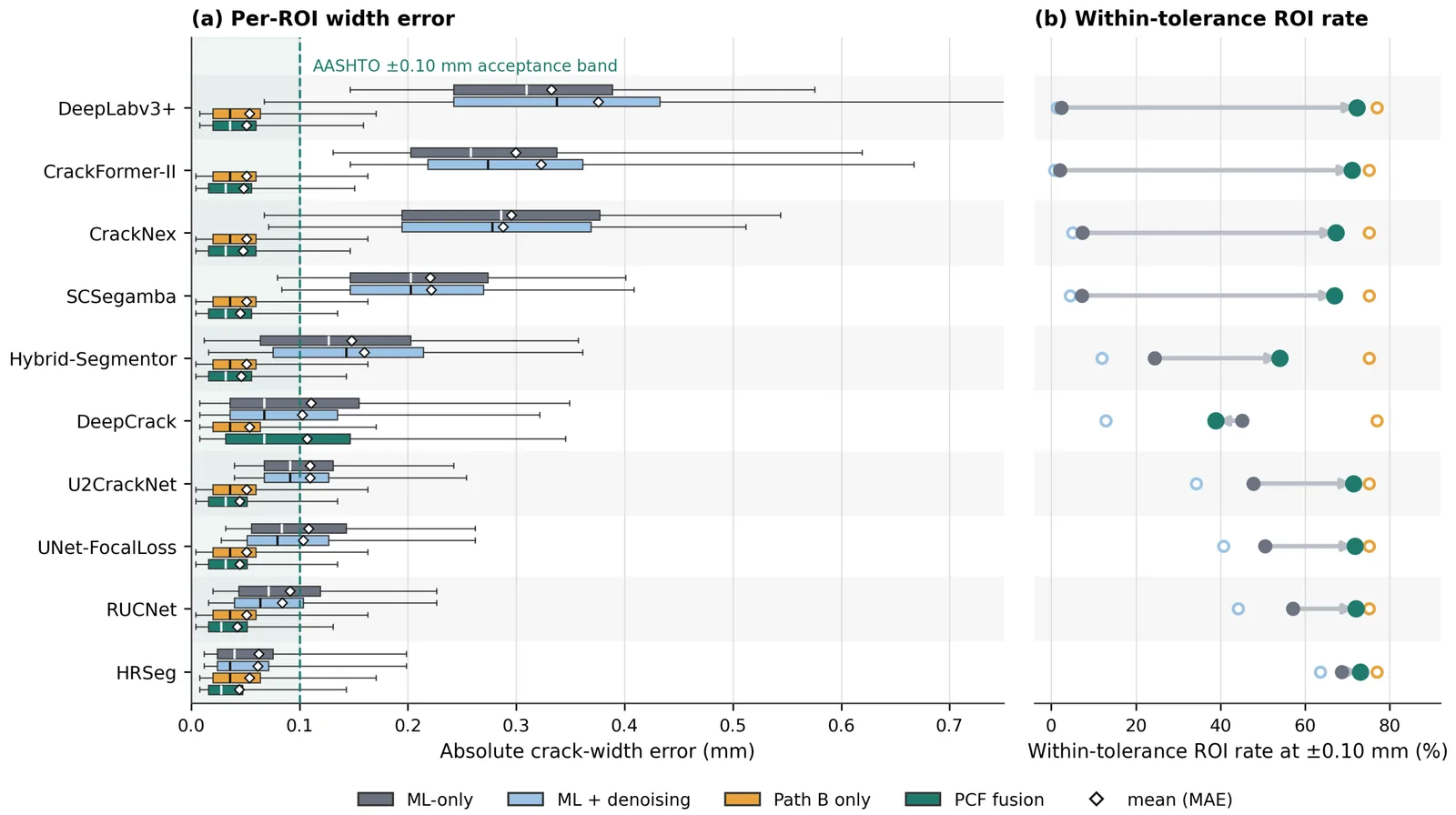
Four-way ablation on all 19,098 krkCMd ROIs. (**a**) Absolute width error; boxes show the interquartile range, whiskers the 5th–95th percentiles, and diamonds the mean. The shaded region marks the ±0.10 mm tolerance. (**b**) Within-tolerance ROI rate. Fusion, not denoising alone, produces the accuracy gain. Path B performs well only when the measurement location is supplied; DeepCrack is the under-segmenting exception.

Figure 8 compares absolute width error and within-tolerance ROI rate across the four arms.

**Denoising alone does not correct the boundary bias.** For the strongest over-segmenters, mean absolute error remains similar or worsens after denoising (DeepLabv3+ 0.333→0.376 mm; CrackFormer-II 0.300→0.323 mm). Even where error decreases slightly, as for RUCNet (0.091→0.084 mm), coverage falls by 20–70 pp. The within-tolerance rate decreases for all ten algorithms.

**Consensus fusion produces the accuracy gain.** For nine of ten algorithms, fusion reduces mean absolute error to 0.042–0.051 mm and raises the within-tolerance rate to 54–73%. For DeepLabv3+, the rate increases by 69.9 pp [+63.8,+75.3] while MAE decreases by 0.281 mm [−0.293,−0.271]. Because the denoising arm uses the same input masks and cleanup stage without Path B intersection, this contrast attributes the gain to consensus fusion.

**Path B alone performs well only when the measurement location is supplied.** At the krkCMd grid lines it reaches a 75–77% within-tolerance rate, but it does not segment the crack globally and also responds to stains, texture, and shadows (Figure 2). Its stand-alone result therefore reflects width estimation at a known location, not autonomous crack measurement. Fusion nevertheless produces lower MAE than Path B alone for nine of ten algorithms (0.042–0.051 vs. 0.051–0.054 mm), consistent with the intended division of labor: Path A localizes the crack, Path B constrains its boundary, and their intersection combines both.

DeepCrack remains the exception. At its external operating point, its under-segmented mask leaves little surplus boundary for intersection to remove, so fusion instead removes true crack pixels. Applying this external configuration to the field set reproduces the failure, reducing coverage from 0.88 to 0.06. At its field operating point (θ=0.40 on RGB input), the same architecture over-segments and benefits from fusion, indicating that the failure is governed by operating point rather than architecture.

### 5.5. H5: The Correction Is Robust to Distance, Tolerance, and Crack Condition

**Capture distance.** On the internal set, the within-tolerance gain is non-negative for every algorithm at all three capture distances, averaging +22, +10, and +11 pp at 0.5, 0.75, and 1.0 m. For nine of ten algorithms, the per-ROI error reduction does not differ significantly across distances (Kruskal–Wallis on paired error deltas, Benjamini–Hochberg adjusted). DeepLabv3+ is the exception, with a larger correction at 0.5 m. Distance therefore changes the magnitude of the benefit but not its direction.

**Figure 9:**
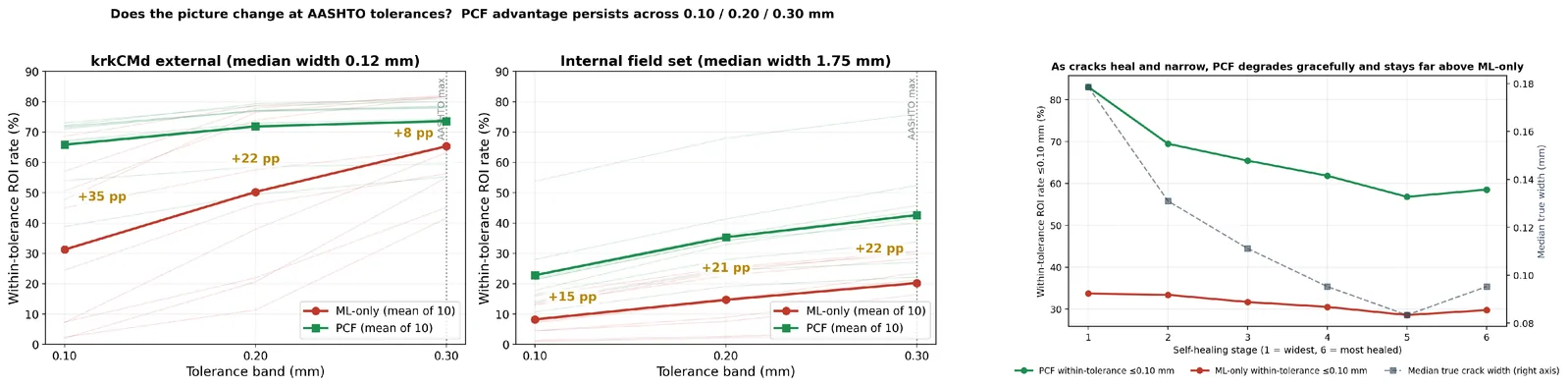
Robustness to tolerance and crack condition. (**Left**): mean within-tolerance ROI rate across ten algorithms at ±0.10/0.20/0.30 mm. PCF retains its advantage on the internal set, while the external methods converge as the tolerance exceeds the scale of the krkCMd cracks. (**Right**): PCF remains above ML-only across the six self-healing stages.

**Tolerance band.** The advantage persists at the looser AASHTO bands (Figure 9, left). On the internal set, the mean gain increases from +15 to +21 to +22 pp at ±0.10/0.20/0.30 mm. On krkCMd, strongly biased algorithms retain substantial gains even at ±0.30 mm (DeepLabv3+ +39 pp), while low-bias algorithms converge with ML-only as the tolerance widens. Because the median krkCMd width is only ≈0.12 mm, the ±0.30 mm band is weakly discriminating on this dataset. Relative tolerances show the same trend: the mean external rate increases from 4.1% to 22.1% at ±10% error and from 13.5% to 41.4% at ±25%.

**Crack condition.** Across the six progressive self-healing stages of krkCMd, PCF maintains an advantage over ML-only as cracks narrow toward the later healing stages (Figure 9, right).

**Along the crack.** Each krkCMd stack carries a median of 86 measurement locations along one crack, so the width profile can be examined rather than only its average. Computed within each crack at a single healing stage, the correlation between measured and reference width rises from +0.428 to +0.838 for DeepLabv3+, improving in 97% of the 206 profiles; nine of ten algorithms converge to +0.754–+0.885 and to a within-crack mean absolute error of 0.036–0.046 mm.

### 5.6. Inferential Validation Across All Ten Algorithms

Paired tests at the ±0.10 mm band confirm the preceding results. PCF significantly lowers absolute width error for eight of ten internal and nine of ten external algorithms (Wilcoxon, H2), and raises the within-tolerance ROI rate for seven of ten internal and nine of ten external algorithms (McNemar, H3). The only significant negative effect is DeepCrack on krkCMd, consistent with its under-segmenting operating point.

**Figure A1:**
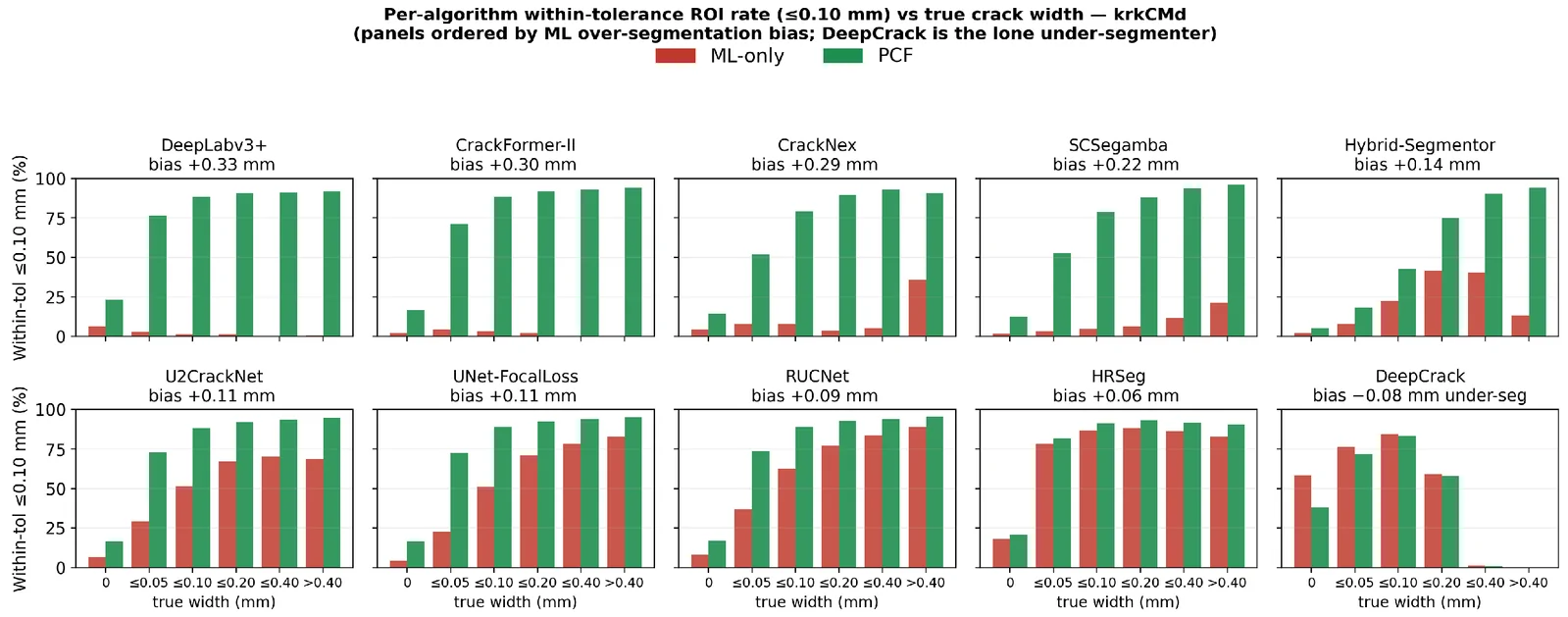
Per-algorithm within-tolerance ROI rate (≤0.10 mm) stratified by true crack width on krkCMd, ordered by ML over-segmentation bias. ML-only (red) vs. PCF (green). High-bias algorithms improve across all widths; HRSeg is already accurate; DeepCrack (under-segmenter) is the inverted, documented failure case.

**Figure A2:**
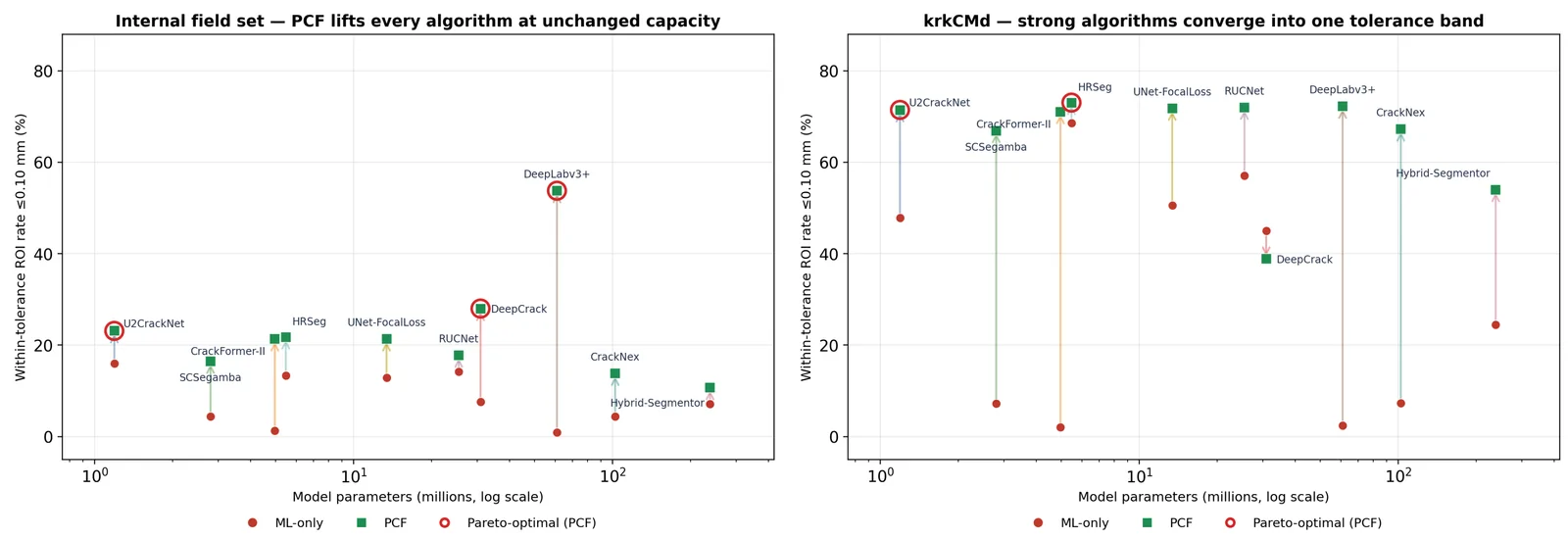
Pareto view of within-tolerance ROI rate (±0.10 mm) versus algorithm capacity on the internal field set (**left**) and external krkCMd set (**right**). PCF (filled) shifts each algorithm’s operating point upward at unchanged capacity relative to ML-only; red circles denote ML-only, green squares denote PCF, arrows connect each algorithm’s two operating points, and red rings mark Pareto-optimal PCF points. Thus, lightweight algorithms approach the heaviest ones; the front collapses toward a common high-yield band.

Appendix A reports the per-algorithm tests and coverage trade; Appendix A.1 and Appendix A.2 provide cluster-robust re-analyses; and Appendix B reports per-algorithm accuracy and the accuracy–capacity comparison (Figure A1 and Figure A2). Qualitative results for all ten algorithms are provided in Appendix D.

## 6. Discussion

Method contribution and scope.

PCF is a lightweight post-processing layer that attaches to existing segmentation algorithms without retraining. Its contribution is a testable correction mechanism: when a segmentation mask systematically over-estimates crack width, intersection with an independent boundary estimate removes surplus pixels and reduces measurement bias. Across ten diverse algorithms, the magnitude and direction of the benefit broadly follow the baseline bias, with consistent gains on both the field and external datasets.

Coverage–accuracy tradeoff.

Intersection slightly reduces ROI coverage because it cannot recover cracks missed by the segmentation model. However, for nine of ten external algorithms this loss is outweighed by a much larger increase in the fraction of ROIs measured within tolerance. This distinction reinforces the need for measurement-based evaluation: coverage indicates whether a width is returned, while the within-tolerance ROI rate indicates whether that width is usable. Likewise, overlap metrics cannot certify physical width accuracy, as shown geometrically in the Introduction and empirically by the directional bias observed in Section 5.1. The dataset-native DLMwidth and AEDwidth estimates provide additional reference points for width measurement at known locations (Appendix B Table A7). We note also that after fusion the 95% limits of agreement span approximately −0.09 to +0.16 mm, wider than the ±0.10 mm band: a large majority of ROIs become usable, not every measurement.

Operating-point dependence.

PCF is most effective when the segmentation algorithm over-segments, and the relevant condition is the operating point rather than the architecture. Model capacity does not predict the bias: across a two-hundred-fold range in parameter count the correlation with external signed bias is r=+0.11. DeepCrack is the boundary case, and its behaviour is sharper than under-segmentation alone. At its external operating point the returned width is statistically independent of the reference (r=−0.05; returned standard deviation 0.032 mm against a reference standard deviation of 0.129 mm), so its apparently competitive 0.111 mm mean absolute error and 45.0% within-tolerance rate are artefacts of a near-constant output falling inside a ±0.10 mm band on a dataset whose median crack is 0.119 mm. There is no width signal for fusion to correct, and intersection removes true crack pixels instead.

Two statistics computed from the pipeline’s output provide exploratory diagnostics of applicability without requiring width references at use time. The shrinkage ratio $R=\mathrm{median}\left(w_{\mathrm{ML}}\right)/\mathrm{median}\left(w_{\mathrm{PCF}}\right)$ is associated with measured bias (r=0.951) and change in within-tolerance rate (r=0.971) across the ten evaluated algorithms. The Path B agreement $A=\mathrm{corr}(w_{\mathrm{ML}},w_{\mathrm{PathB}})$ is 0.474–0.905 for the nine algorithms helped by fusion and −0.003 for DeepCrack, for which fusion lowers the within-tolerance rate by 6.2 percentage points. Thus, *A* separates the beneficial and adverse operating points in this evaluated set. With only ten algorithm-level cases from one dataset and one adverse case, this is preliminary evidence rather than a validated deployment rule for unseen models, domains or individual images.

A supervised algorithm can only learn the notion of width encoded in its annotations, and hand-drawn boundaries are not exact: read at the same probe points with the same estimator, our own ground-truth masks give a median width of 1.456 mm against a physical 1.750 mm. Annotation therefore places a ceiling on width fidelity that better segmentation alone cannot lift, which is a further argument for supplying boundary evidence taken from the image rather than from a label. Attributing the remaining differences between algorithms to receptive field, loss family or training regime would require controlled retraining across matched corpora and objectives, which is beyond the scope of this study.

Limitations.

The external dataset contains 19,098 ROIs but only 36 image stacks from a single self-healing-concrete material, limiting material and texture diversity despite the use of stack-clustered confidence intervals. Its very high native resolution (9448×6305 px) also means that even sub-millimeter cracks span many pixels, so performance on lower-resolution imagery remains to be characterized. Finally, PCF remains a calibrated procedure: its operating points were selected on the internal field dataset and transferred unchanged to krkCMd, but broader cross-domain validation is needed to determine how well those settings generalize to other cameras, materials, and acquisition conditions. Four further limits apply. The field reference is a single caliper point per ROI taken by one operator, so the internal comparison tests point agreement rather than profile agreement and no inter-observer reliability is established. Shadows, staining and branching are present in the field imagery but unannotated, so their effect on accuracy is not quantified. A full photogrammetric treatment of tilt, lens distortion and depth of field is not attempted. ML-only and PCF use the same images, ROIs and per-image scale factors, so their comparison holds acquisition and calibration procedures fixed. Nevertheless, shared calibration does not eliminate geometric uncertainty: residual distortion may affect absolute widths, error magnitudes and classification against the ±0.10 mm tolerance. The paired design supports comparison under the same acquisition conditions, but does not establish immunity to geometric error. Finally, detection is not benchmarked end to end over complete scenes. Coverage reports the fraction of supplied reference locations at which a width is returned; it is not complete-scene detection precision or recall. Neither dataset provides the exhaustive crack annotations needed for that benchmark.

## 7. Conclusions

This paper presented Parallel Consensus Fusion (PCF), a calibrated measurement-reliability layer that corrects crack-width over-segmentation without retraining, applicable to any segmentation algorithm whose operating point over-segments. PCF intersects a learned segmentation mask with an independent contrast-based boundary estimate before width extraction. We evaluated it across ten architecturally diverse segmentation algorithms on an uncontrolled field dataset containing 75 physical ROIs observed at three capture distances (225 image–ROI observations) and an external dataset of 19,098 operator-measured ROIs. PCF operating points were calibrated on the internal field data and transferred unchanged to the external evaluation.

The results support a conditional benefit of PCF at over-segmenting operating points, with the strength of evidence varying by algorithm and dataset. On the external set, nine of ten algorithms exhibited positive width bias, with width over-estimated at 88.7–99.8% of ROIs, confirming that segmentation-derived width error is predominantly directional rather than random. Consensus fusion reduced this bias and lowered absolute width error; on krkCMd, nine algorithms converged to 0.042–0.051 mm mean absolute error despite spanning 1.2–238 M parameters. At the strict ±0.10 mm criterion, PCF increased the within-tolerance ROI rate for all ten algorithms internally and nine of ten externally. A four-way ablation showed that the improvement arises from consensus fusion rather than denoising alone, while Path B’s strong known-location measurements do not translate to independent crack segmentation. DeepCrack, the sole under-segmenting operating point, provided the predicted failure case. The benefit also persisted across capture distance, wider tolerance bands, and progressive crack-healing conditions.

These findings show that high segmentation overlap does not by itself establish crack-width reliability. For inspection-oriented measurement, performance should instead reflect whether a width is both returned and sufficiently accurate; the within-tolerance ROI rate provides that criterion. PCF offers a lightweight way to improve that reliability across otherwise diverse segmentation models while making its principal limitation explicit: it is effective when the baseline segmentation exhibits positive width bias.

## 8. Future Work

Future validation should focus primarily on domain generalization. Although PCF was evaluated across ten CNN, transformer, Mamba, and hybrid segmentation algorithms, the external dataset contains only one self-healing-concrete material. Additional width-labeled datasets spanning materials, surface textures, cameras, resolutions, and field conditions are needed to establish how broadly the observed over-segmentation mechanism and calibrated operating points transfer.

PCF also remains a calibrated procedure. Automated or adaptive selection of its operating point from model confidence, image statistics, or a small in-situ calibration target could reduce manual calibration and improve deployment across new acquisition conditions.

PCF currently corrects positive width bias but cannot recover pixels from an under-segmented mask. A complementary dilation- or union-based fusion mode, selected from the sign of the baseline width bias, could extend the framework to both over- and under-segmentation.

Finally, integration into field AR systems introduces deployment questions beyond measurement accuracy. In our HoloLens 2 prototype, the headset captures and displays results while a paired laptop performs segmentation and PCF over Wi-Fi. Future work will evaluate round-trip latency, throughput, and usability during active inspection; current processing costs are reported in Appendix H.

## Figures and Tables

**Table 1:** PCF operating points, and the data on which each was selected. All parameters were calibrated on the internal field dataset and held fixed for subsequent evaluation, including external testing on krkCMd.

Parameter or Stage	Value	Selected on	Reference Used
Path A threshold, DeepLabv3+/DeepCrack	θ=0.85/0.40	Internal field set	Caliper widths
Path A, remaining eight algorithms	Default released settings	Not tuned	None
Path B grayscale threshold	15 (established)/30 (modern)	Internal field set, coverage-constrained grid	Caliper widths
CLAHE clip and tile	2.0, 15×15/4.0, 8×8	Internal field set, same grid	Caliper widths
Fusion mode	Intersection	Fixed by design	None
Stage 3 post-fusion filters	Fixed constants	Internal field set	Caliper widths
Stage 4 width estimator	Medial-axis run, normal sampling	Internal ROIs, 38-variant benchmark	GT masks and caliper widths
Internal evaluation	225 image–ROI observations (75 ROIs, 15 cracks)	—	Caliper widths
External evaluation	19,098 ROIs (36 stacks)	—	Operator widths, evaluation only

**Figure A3:**
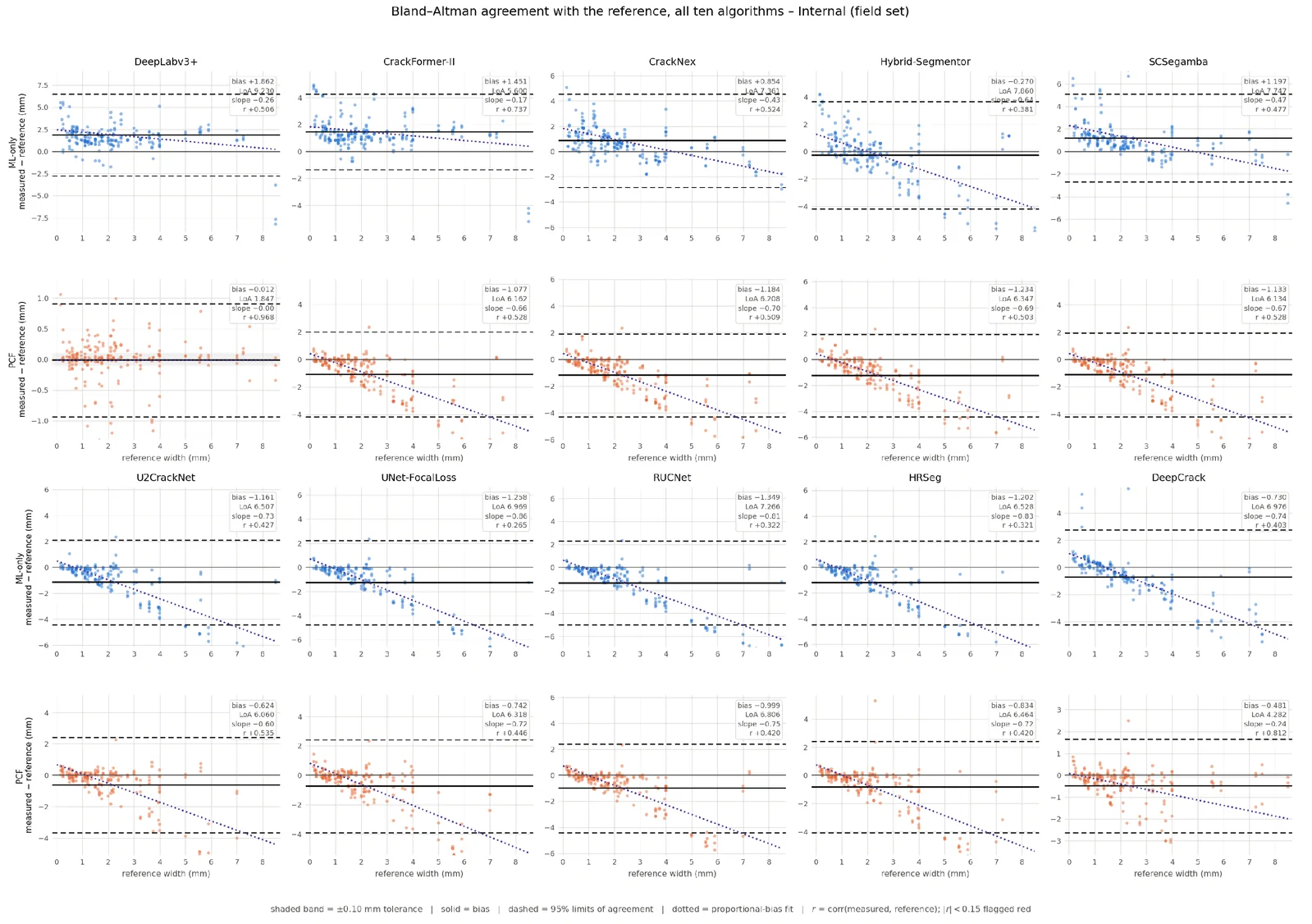
Agreement with the caliper reference, all ten algorithms, field set. Blue points, ML-only (upper row of each algorithm pair); orange points, PCF (lower row). Solid line, mean difference (bias); dashed lines, 95% limits of agreement; dotted line, proportional-bias fit; shaded band, the ±0.10 mm tolerance.

**Figure A4:**
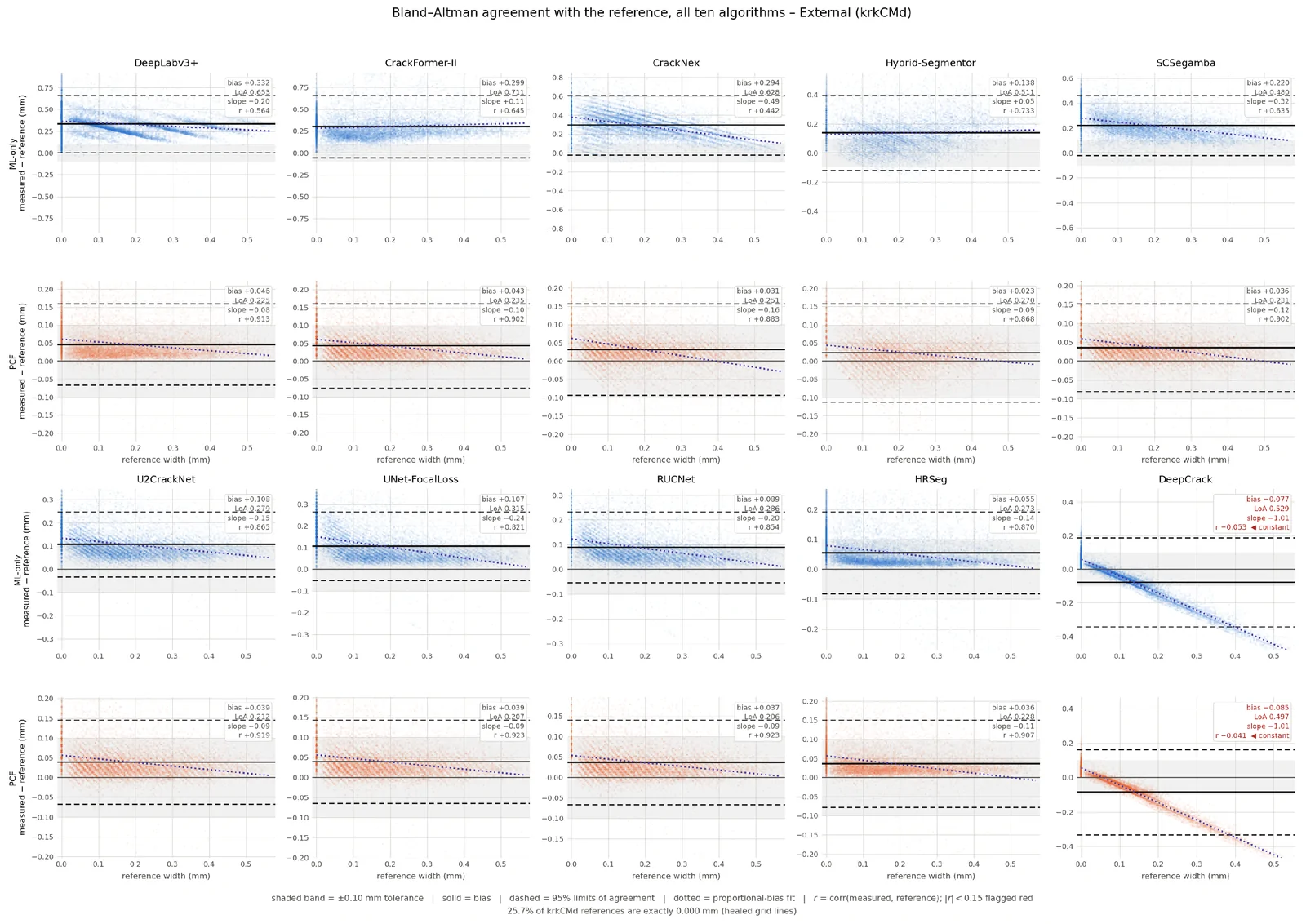
Agreement with the operator reference, all ten algorithms, krkCMd. Blue points, ML-only (upper row of each algorithm pair); orange points, PCF (lower row). Solid line, mean difference (bias); dashed lines, 95% limits of agreement; dotted line, proportional-bias fit; shaded band, the ±0.10 mm tolerance.

**Figure A5:**
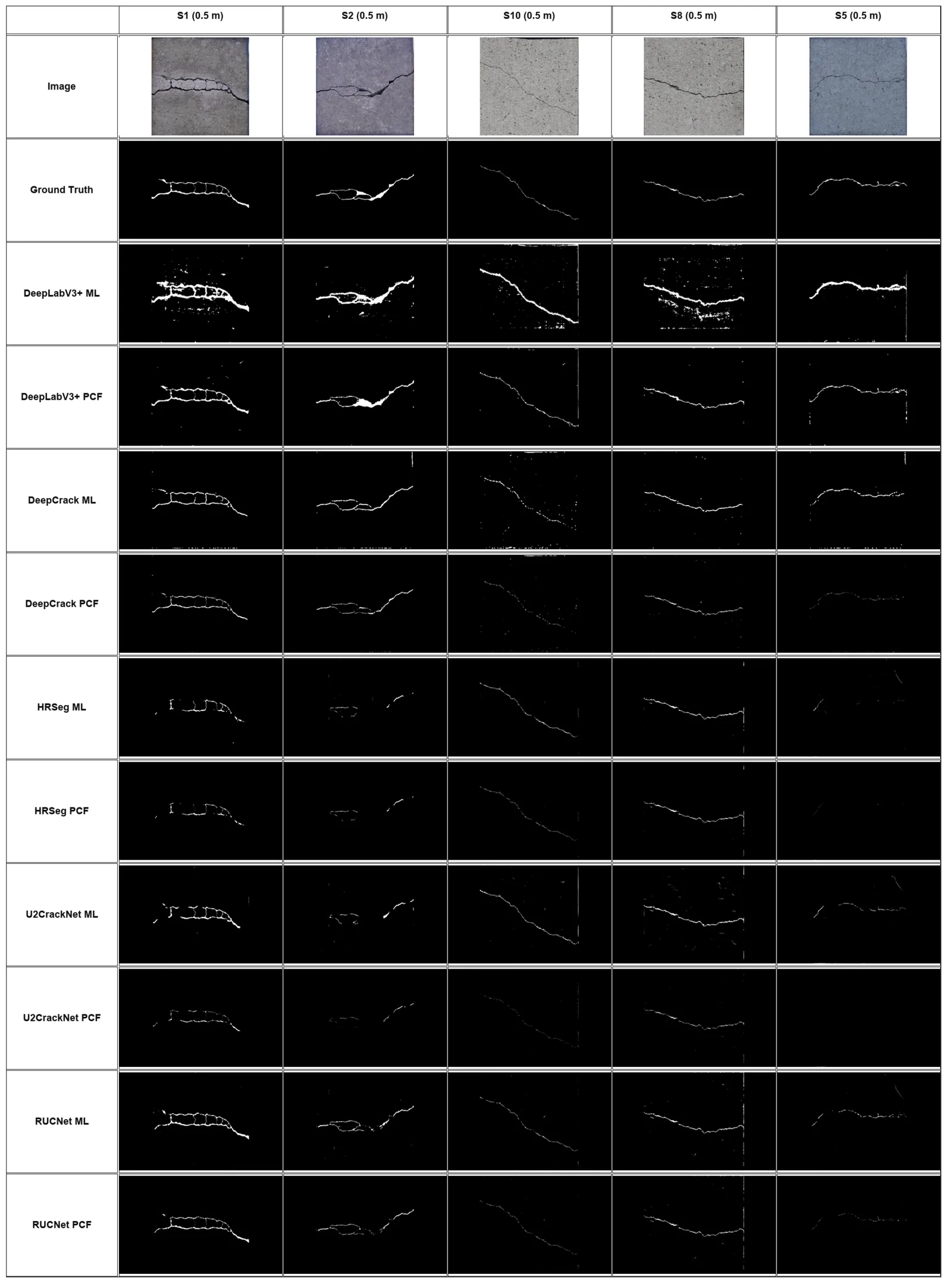
Qualitative examples (1 of 2): ML-only vs. PCF masks for DeepLabv3+, DeepCrack, HRSeg, U2CrackNet, and RUCNet on five internal samples (0.5 m distance), with input image and ground truth in the top rows.

**Figure A6:**
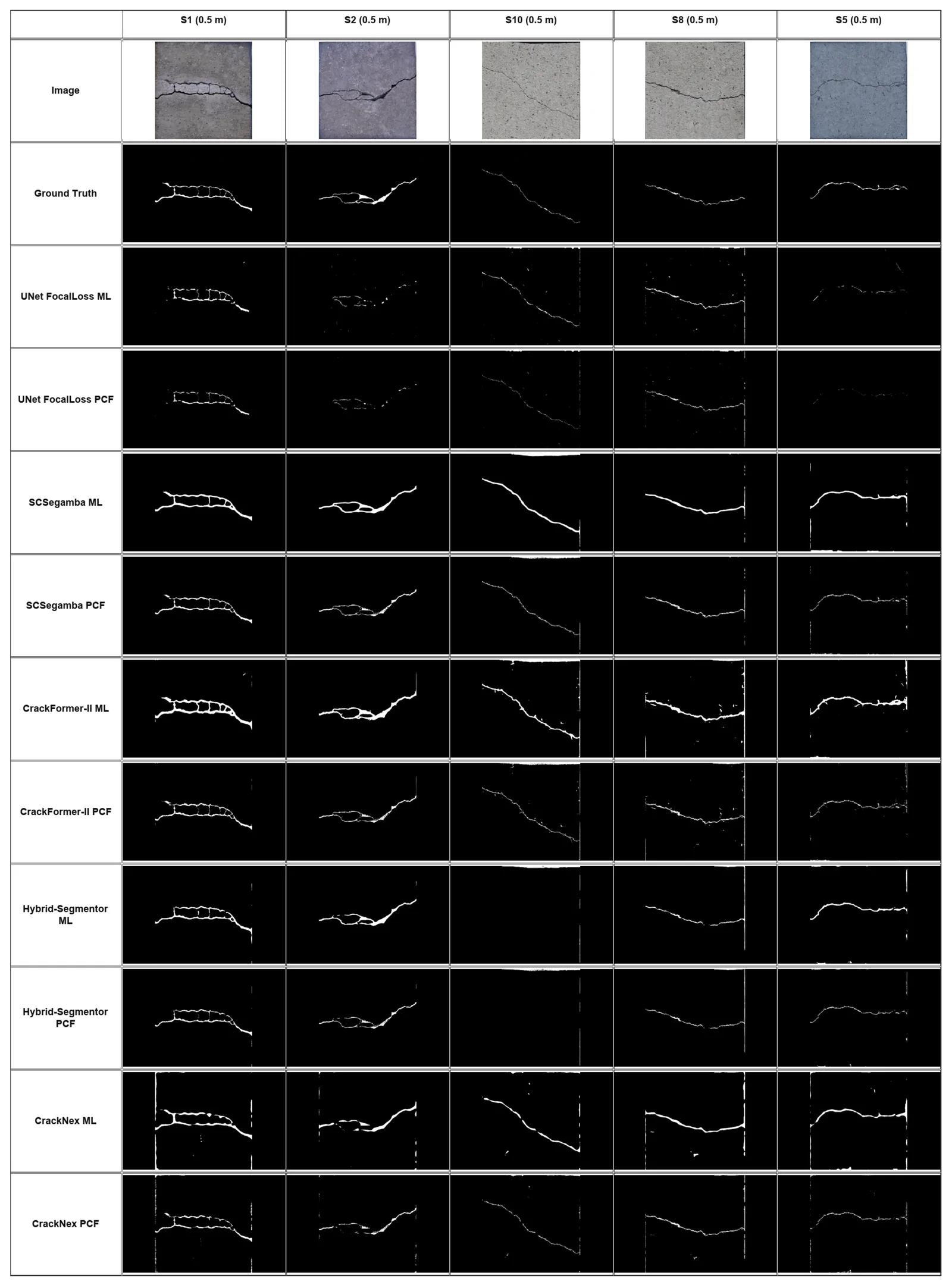
Qualitative examples (2 of 2): ML-only vs. PCF masks for UNet-FocalLoss, SCSegamba, CrackFormer-II, Hybrid-Segmentor, and CrackNex on the same five internal samples.

**Figure A7:**
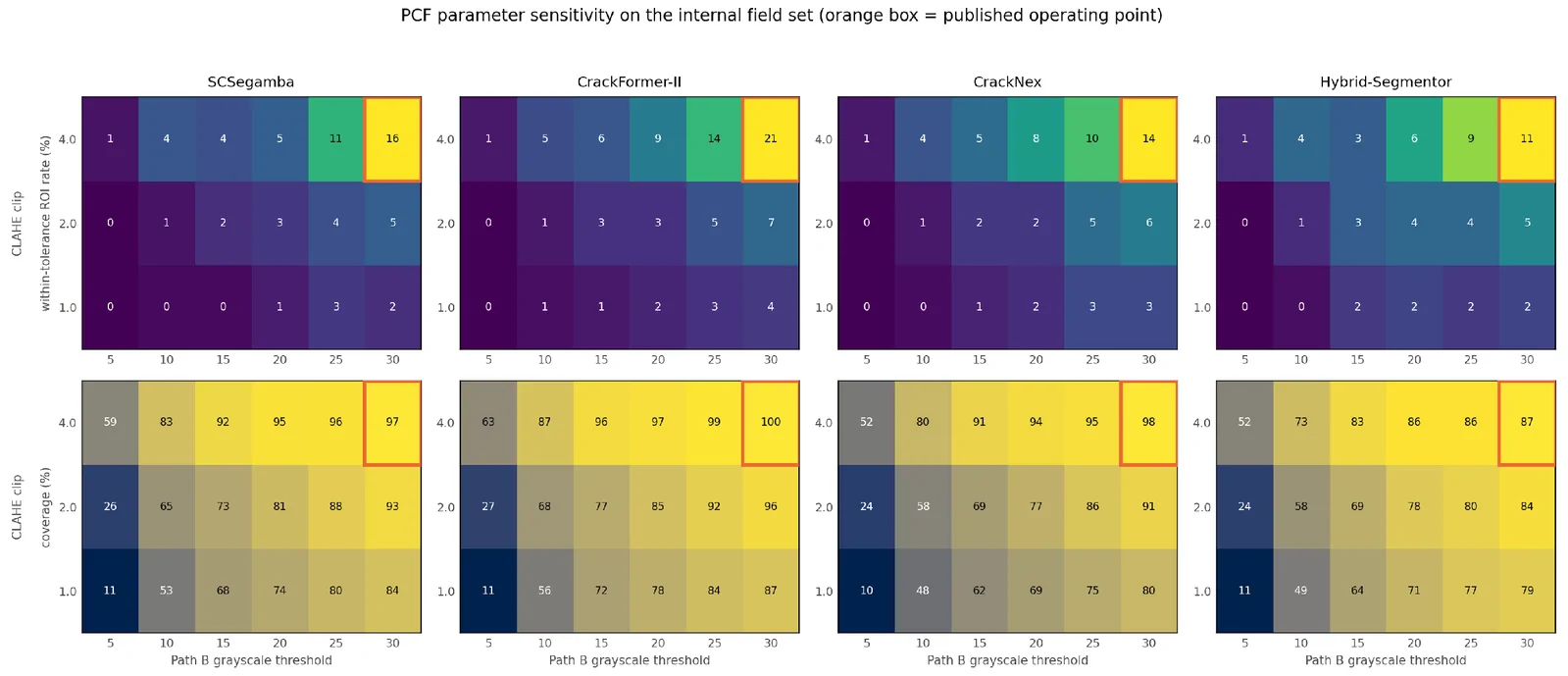
PCF parameter sensitivity on the internal field set. Top row, within-tolerance ROI rate; bottom row, coverage. Cell color encodes the value printed in the cell, from dark (low) to bright (high), with an independent scale for each row; it carries no information beyond the printed numbers. The published operating point is boxed.

**Figure A8:**
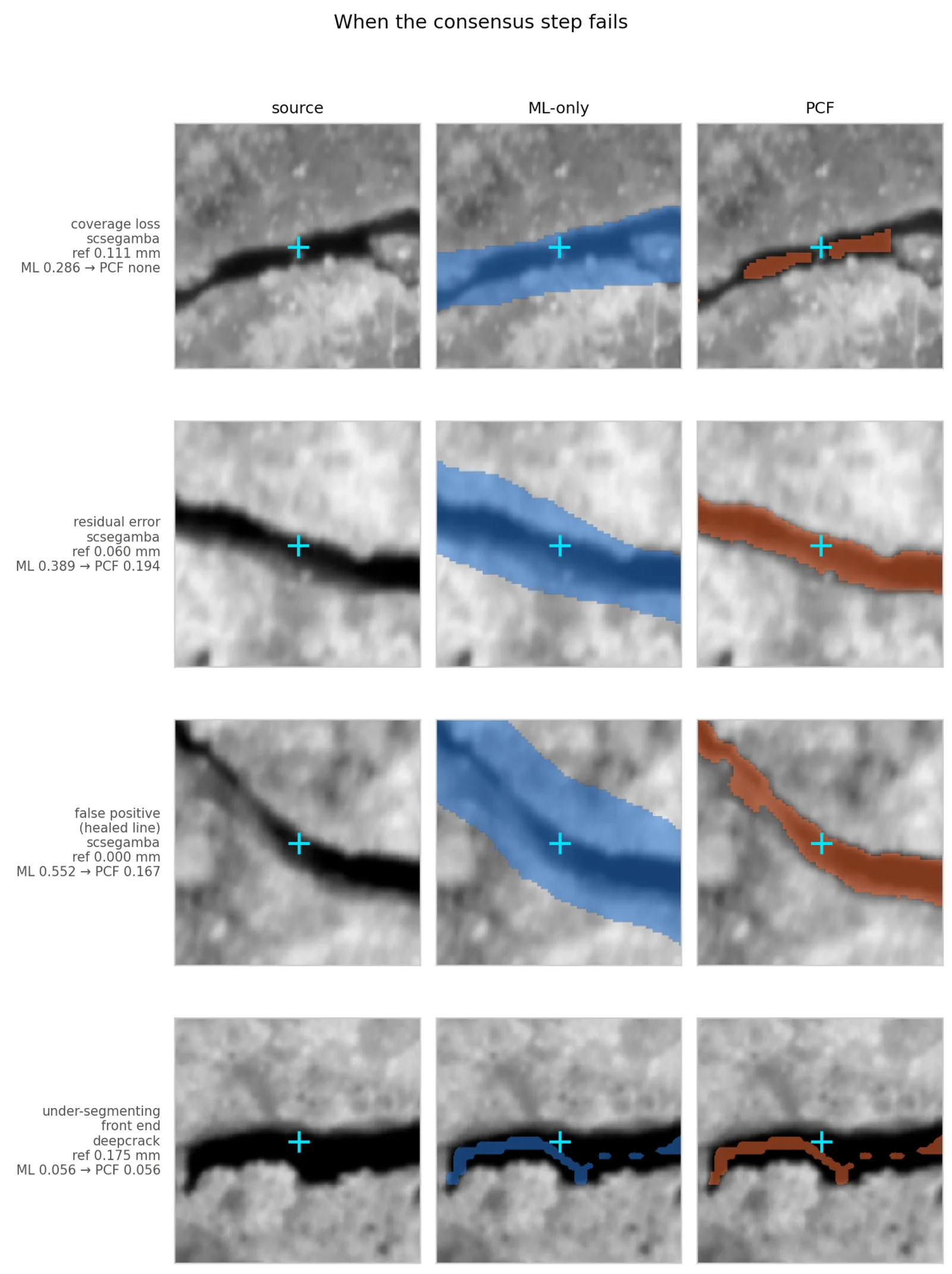
Failure modes on krkCMd: coverage loss, where the consensus mask follows the crack more closely than the learned mask but breaks at the measurement point; residual error, where fusion halves a six-fold over-segmentation but remains above tolerance; a false positive on a grid line recorded as healed; and an under-segmenting front end, where no surplus boundary remains to trim.

## Data Availability

The external krkCMd dataset analyzed in this study is publicly available from its original publication. The field dataset generated and analyzed in this study is available from https://doi.org/10.7294/29361029.

## Appendix A. Inferential Statistics Across All Algorithms and Both Datasets

This appendix reports the per-algorithm hypothesis tests for all ten models on the internal (N=225 image–ROI observations) and external (N=19,098 ROIs) datasets. H2 uses paired Wilcoxon signed-rank tests on absolute error for ROIs measurable by both methods; H3 uses McNemar’s test on paired within-tolerance outcomes over all ROIs. Within each hypothesis and dataset, *p*-values are Benjamini–Hochberg adjusted across the ten algorithms. The primary tolerance is ±0.10 mm; results at ±0.20 and ±0.30 mm are provided in Table A4.

**Table A1:** H2 (absolute-error reduction): paired Wilcoxon signed-rank test on |e| (mm). $\Delta =\mathrm{median}(|e_{\mathrm{PCF}}|-|e_{\mathrm{ML}}|)$; negative favors PCF. $p_{\mathrm{BH}}$ is Benjamini–Hochberg adjusted across the ten algorithms. ROI-level; see Table A5 for the cluster-robust external re-analysis.

Algorithm	Paired *n*	Median $|e_{\mathrm{ML}}|$	Median $|e_{\mathrm{PCF}}|$	Median Δ	$p_{\mathrm{BH}}$
Internal field set					
U2CrackNet	185	0.552	0.281	−0.120	$2.2\times 10^{-12}$
SCSegamba	219	0.935	0.583	−0.179	0.158
CrackFormer-II	225	1.268	0.458	−0.756	$1.7\times 10^{-5}$
HRSeg	182	0.617	0.330	−0.079	$2.2\times 10^{-12}$
UNet-FocalLoss	185	0.639	0.330	−0.111	$4.1\times 10^{-10}$
RUCNet	193	0.709	0.380	−0.076	$1.6\times 10^{-13}$
DeepCrack	197	0.663	0.281	−0.279	$3.7\times 10^{-8}$
DeepLabv3+	222	1.601	0.084	−1.374	$4.7\times 10^{-36}$
CrackNex	220	0.865	0.800	+0.009	0.706
Hybrid-Segmentor	196	0.831	0.825	+0.000	0.856
External krkCMd set					
U2CrackNet	14,962	0.091	0.032	−0.056	<$10^{-300}$
SCSegamba	14,037	0.198	0.032	−0.159	<$10^{-300}$
CrackFormer-II	15,114	0.258	0.032	−0.214	<$10^{-300}$
HRSeg	15,408	0.036	0.028	−0.008	<$10^{-300}$
UNet-FocalLoss	15,027	0.079	0.032	−0.048	<$10^{-300}$
RUCNet	14,988	0.067	0.028	−0.032	<$10^{-300}$
DeepCrack	11,435	0.067	0.067	+0.000	$1.3\times 10^{-60}$
DeepLabv3+	15,562	0.310	0.036	−0.266	<$10^{-300}$
CrackNex	14,287	0.286	0.032	−0.242	<$10^{-300}$
Hybrid-Segmentor	11,439	0.127	0.032	−0.087	<$10^{-300}$

**Table A2:** Coverage: McNemar’s test on paired measurable/NaN outcomes. “Recovered” = ML miss → PCF valid; “Lost” = ML valid → PCF miss. ARR = PCF coverage − ML coverage. Because the consensus mask is an intersection, on krkCMd PCF cannot recover an ROI the segmentation algorithm missed, so it trades raw coverage (negative ARR) for the large accuracy gains in Table A1 and Table A3.

Algorithm	*n*	Recovered	Lost	$p_{\mathrm{BH}}$	ARR (95% CI)
Internal field set					
U2CrackNet	225	16	0	$7.6\times 10^{-5}$	+0.071[+0.038,+0.105]
SCSegamba	225	0	2	0.625	−0.009[−0.021,+0.003]
CrackFormer-II	225	0	0	1.00	+0.000[+0.000,+0.000]
HRSeg	225	24	0	$1.2\times 10^{-6}$	+0.107[+0.066,+0.147]
UNet-FocalLoss	225	19	0	$1.9\times 10^{-5}$	+0.084[+0.048,+0.121]
RUCNet	225	17	0	$5.1\times 10^{-5}$	+0.076[+0.041,+0.110]
DeepCrack	225	23	4	$1.1\times 10^{-3}$	+0.084[+0.041,+0.128]
DeepLabv3+	225	0	1	1.00	−0.004[−0.013,+0.004]
CrackNex	225	0	4	0.208	−0.018[−0.035,−0.001]
Hybrid-Segmentor	225	0	3	0.357	−0.013[−0.028,+0.002]
External krkCMd set					
U2CrackNet	19,098	0	996	$7.2\times 10^{-218}$	−0.052[−0.055,−0.049]
SCSegamba	19,098	0	768	$1.7\times 10^{-168}$	−0.040[−0.043,−0.037]
CrackFormer-II	19,098	0	939	$1.3\times 10^{-205}$	−0.049[−0.052,−0.046]
HRSeg	19,098	0	394	$3.4\times 10^{-87}$	−0.021[−0.023,−0.019]
UNet-FocalLoss	19,098	0	1038	$6.7\times 10^{-227}$	−0.054[−0.058,−0.051]
RUCNet	19,098	0	993	$2.7\times 10^{-217}$	−0.052[−0.055,−0.049]
DeepCrack	19,098	0	2046	<$10^{-300}$	−0.107[−0.112,−0.103]
DeepLabv3+	19,098	0	1625	<$10^{-300}$	−0.085[−0.089,−0.081]
CrackNex	19,098	0	1567	<$10^{-300}$	−0.082[−0.086,−0.078]
Hybrid-Segmentor	19,098	0	337	$7.8\times 10^{-75}$	−0.018[−0.020,−0.016]

**Table A3:** H3 (tolerance compliance at ±0.10 mm): McNemar’s test on paired pass/fail. “No→Yes” = ML fail to PCF pass; “Yes→No” = the reverse. ARR = PCF pass rate − ML pass rate; NNT =1/ARR. For krkCMd we also give a cluster-bootstrap 95% CI on ARR that resamples the 36 image stacks (effective sample size), since the naive McNemar treats the 19,098 ROIs as independent.

Algorithm	*n*	No→Yes	Yes→No	$p_{\mathrm{BH}}$	ARR	NNT	ARR 95% CI (Clustered)
Internal field set							
U2CrackNet	225	35	19	0.052	+0.071	14.1	–
SCSegamba	225	36	9	$2.7\times 10^{-4}$	+0.120	8.3	–
CrackFormer-II	225	48	3	$3.6\times 10^{-9}$	+0.200	5.0	–
HRSeg	225	32	13	0.010	+0.084	11.8	–
UNet-FocalLoss	225	32	13	0.010	+0.084	11.8	–
RUCNet	225	22	14	0.243	+0.036	28.1	–
DeepCrack	225	58	12	$2.5\times 10^{-7}$	+0.204	4.9	–
DeepLabv3+	225	121	2	$1.9\times 10^{-25}$	+0.529	1.9	–
CrackNex	225	31	10	$3.6\times 10^{-3}$	+0.093	10.7	–
Hybrid-Segmentor	225	20	12	0.240	+0.036	28.1	–
External krkCMd set							
U2CrackNet	19,098	4874	358	<$10^{-300}$	+0.236	4.2	+0.184,+0.293
SCSegamba	19,098	11,524	123	<$10^{-300}$	+0.597	1.7	+0.530,+0.659
CrackFormer-II	19,098	13,288	106	<$10^{-300}$	+0.690	1.4	+0.626,+0.751
HRSeg	19,098	1165	315	$6.3\times 10^{-108}$	+0.045	22.5	+0.023,+0.068
UNet-FocalLoss	19,098	4334	283	<$10^{-300}$	+0.212	4.7	+0.160,+0.268
RUCNet	19,098	3253	411	<$10^{-300}$	+0.149	6.7	+0.102,+0.200
DeepCrack	19,098	133	1311	$1.4\times 10^{-210}$	−0.062	–	−0.082,−0.045
DeepLabv3+	19,098	13,649	307	<$10^{-300}$	+0.699	1.4	+0.638,+0.753
CrackNex	19,098	11,884	444	<$10^{-300}$	+0.599	1.7	+0.542,+0.655
Hybrid-Segmentor	19,098	5832	195	<$10^{-300}$	+0.295	3.4	+0.222,+0.366

**Table A4:** Sanity check: H3 trend across tolerances. “Sig. positive” counts algorithms with a BH-significant ($p_{\mathrm{BH}}<0.05$) positive ARR; “Negative ARR” counts algorithms for which PCF lowers the pass rate. The ±0.10 mm conclusion holds at ±0.20 and ±0.30 mm: on the internal set the effect strengthens, while on krkCMd the already-low-bias algorithms converge with ML-only as the band widens past the sub-millimetre crack sizes.

Dataset	τ (mm)	Sig. Positive	Negative ARR	Median ARR
Internal	0.10	7/10	0/10	+0.089
Internal	0.20	9/10	0/10	+0.140
Internal	0.30	9/10	0/10	+0.153
krkCMd	0.10	9/10	1/10	+0.266
krkCMd	0.20	6/10	3/10	+0.078
krkCMd	0.30	5/10	5/10	+0.009

### Appendix A.1. Cluster-Robust Re-Analysis (External krkCMd)

Because the 19,098 external ROIs are nested within 36 image stacks, Table A5 repeats the H2 and H3 analyses with stack-level clustering. Confidence intervals use a cluster bootstrap over the 36 stacks; H2 uses a linear mixed model with a random stack intercept, and H3 a GEE logistic model with exchangeable within-stack correlation. The table reports the median paired error reduction with its bootstrap interval, while the H2 *p*-value tests the mean paired reduction. Both estimands agree in direction for every algorithm. PCF significantly improves both outcomes for nine of ten algorithms, with DeepCrack remaining the negative exception.

**Table A5:** Cluster-robust external results. H2: paired median reduction in |e| (mm), negative favours PCF; H3: within-tolerance ARR at ±0.10 mm, positive favours PCF. Brackets are 95% cluster-bootstrap intervals over the 36 stacks; *p* is the cluster-aware test (mixed model for H2, GEE for H3).

Algorithm	H2 Median Δ|e| [95% CI]	H2 *p*	H3 ARR [95% CI]	H3 *p*
DeepLabv3+	−0.266[−0.286,−0.258]	$3.0\times 10^{-61}$	+0.699[+0.627,+0.781]	$4.0\times 10^{-30}$
CrackFormer-II	−0.214[−0.226,−0.206]	$1.4\times 10^{-27}$	+0.690[+0.616,+0.779]	$4.1\times 10^{-21}$
CrackNex	−0.242[−0.270,−0.218]	$2.7\times 10^{-34}$	+0.599[+0.555,+0.657]	<$10^{-16}$
SCSegamba	−0.159[−0.163,−0.159]	$1.2\times 10^{-27}$	+0.597[+0.508,+0.707]	$4.9\times 10^{-33}$
Hybrid-Segmentor	−0.087[−0.111,−0.071]	$6.8\times 10^{-30}$	+0.295[+0.204,+0.443]	$1.9\times 10^{-8}$
U2CrackNet	−0.056[−0.067,−0.048]	$7.1\times 10^{-29}$	+0.236[+0.173,+0.379]	$1.4\times 10^{-6}$
UNet-FocalLoss	−0.048[−0.064,−0.036]	$9.6\times 10^{-20}$	+0.212[+0.149,+0.359]	$2.8\times 10^{-6}$
RUCNet	−0.032[−0.056,−0.032]	$5.0\times 10^{-13}$	+0.149[+0.090,+0.285]	$4.0\times 10^{-4}$
HRSeg	−0.008[−0.016,−0.004]	$5.9\times 10^{-4}$	+0.045[+0.016,+0.103]	0.037
DeepCrack	+0.000[+0.000,+0.000]	0.048	−0.062[−0.079,−0.032]	$9.5\times 10^{-11}$

### Appendix A.2. Cluster-Robust Re-Analysis (Internal Field Set)

Because the 225 internal image–ROI observations are nested within 15 physical cracks, Table A6 repeats the H2 and H3 analyses using the crack as the resampling unit, with 95% cluster-bootstrap intervals over the 15 cracks. The H3 gain is positive for all ten algorithms and excludes zero for seven; RUCNet, U2CrackNet, and Hybrid-Segmentor remain positive but cross zero. For H2, error reductions remain strongest for the more biased algorithms and approach zero for those with lower baseline error, consistent with the main results.

**Table A6:** Cluster-robust internal results. H2: paired median change in |e| (mm), negative favours PCF; H3: within-tolerance ARR at ±0.10 mm, positive favours PCF. Brackets are 95% cluster-bootstrap intervals over the 15 physical cracks.

Algorithm	H2 Median Δ|e| [95% CI]	H3 ARR [95% CI]
DeepLabv3+	−1.374[−1.659,−1.223]	+0.529[+0.476,+0.587]
DeepCrack	−0.308[−0.416,−0.087]	+0.204[+0.107,+0.324]
CrackFormer-II	−0.756[−1.006,−0.252]	+0.200[+0.102,+0.298]
SCSegamba	−0.179[−0.757,+0.124]	+0.120[+0.027,+0.200]
CrackNex	+0.009[−0.384,+0.297]	+0.093[+0.009,+0.182]
HRSeg	−0.079[−0.190,+0.000]	+0.084[+0.022,+0.151]
UNet-FocalLoss	−0.111[−0.220,+0.000]	+0.084[+0.018,+0.156]
U2CrackNet	−0.120[−0.260,−0.039]	+0.071[−0.013,+0.160]
RUCNet	−0.076[−0.138,+0.000]	+0.036[−0.013,+0.089]
Hybrid-Segmentor	+0.000[+0.000,+0.073]	+0.036[−0.013,+0.089]

### Appendix A.3. Leave-One-Crack-Out Calibration Check

To test whether the shared modern-family operating point depends on any individual crack, we repeated the internal calibration in a leave-one-crack-out protocol. In each of 15 folds, the coverage-constrained search over six grayscale thresholds and three CLAHE clip limits was performed on 14 cracks and evaluated on the held-out crack. The published configuration (threshold 30, CLAHE clip 4.0, tile 8) was selected in all 15 folds. The pooled held-out performance was therefore identical to that obtained with the full internal calibration (within-tolerance ROI rate 15.6% at ±0.10 mm; MAE 1.254 mm), indicating that the selected operating point is not driven by any single crack.

## Appendix B. Per-Algorithm Accuracy, Dataset References, and the Pareto View

This section reports the per-algorithm accuracy underlying the main-text summaries and, for krkCMd, the dataset-native DLMwidth and AEDwidth estimates. These are width measurements at supplied grid-line locations and do not perform crack detection or segmentation, so they are reported as reference points rather than direct comparators. Within-tolerance ROI rates use the primary ±0.10 mm criterion. The final column, *r*, is the correlation between the returned width and the reference; a value near zero indicates a pipeline whose output does not track crack width, which no mean-based summary reveals.

**Table A7:** External krkCMd: per-algorithm width accuracy (ML-only vs. PCF) alongside the dataset’s profile-based width references (DLMwidth, AEDwidth), which read width at a supplied location and are reference points rather than comparators (see External Validation Datasets). Coverage and the within-tolerance ROI rate are percentages of all 19,098 ROIs.

Algorithm	Variant	Params (M)	Coverage (%)	MAE (mm)	Within-tol. (%)	*r*
DLMwidth	reference (DL)	—	100.0	0.014	98.6	—
AEDwidth	reference (analytic)	—	100.0	0.034	95.3	—
DeepLabv3+	ML-only	61.0	90.0	0.333	2.4	+0.56
DeepLabv3+	PCF	61.0	81.5	0.051	72.2	+0.91
DeepCrack	ML-only	30.9	70.6	0.111	45.0	−0.05
DeepCrack	PCF	30.9	59.9	0.107	38.9	−0.04
HRSeg	ML-only	5.5	82.7	0.062	68.6	+0.87
HRSeg	PCF	5.5	80.7	0.044	73.0	+0.91
RUCNet	ML-only	25.5	83.7	0.091	57.1	+0.85
RUCNet	PCF	25.5	78.5	0.042	72.0	+0.92
U2CrackNet	ML-only	1.2	83.6	0.109	47.8	+0.87
U2CrackNet	PCF	1.2	78.3	0.045	71.5	+0.92
UNet-FocalLoss	ML-only	13.4	84.1	0.108	50.6	+0.82
UNet-FocalLoss	PCF	13.4	78.7	0.045	71.8	+0.92
SCSegamba	ML-only	2.8	77.5	0.221	7.2	+0.64
SCSegamba	PCF	2.8	73.5	0.045	66.9	+0.90
CrackFormer-II	ML-only	5.0	84.1	0.300	2.0	+0.65
CrackFormer-II	PCF	5.0	79.1	0.048	71.1	+0.90
CrackNex	ML-only	102.3	83.0	0.295	7.3	+0.44
CrackNex	PCF	102.3	74.8	0.048	67.2	+0.88
Hybrid-Segmentor	ML-only	237.9	61.7	0.148	24.5	+0.73
Hybrid-Segmentor	PCF	237.9	59.9	0.046	54.0	+0.87

**Table A8:** Internal field set: per-algorithm width accuracy (ML-only vs. PCF) over the 225 probe ROIs. The within-tolerance ROI rate is evaluated at ±0.10 mm.

Algorithm	Variant	Coverage (%)	MAE (mm)	Median |e| (mm)	Within-tol. (%)	*r*
DeepLabv3+	ML-only	99.1	2.109	1.593	0.9	+0.51
DeepLabv3+	PCF	98.7	0.247	0.084	53.8	+0.97
DeepCrack	ML-only	89.3	1.212	0.655	7.6	+0.40
DeepCrack	PCF	97.8	0.608	0.271	28.0	+0.81
HRSeg	ML-only	80.9	1.269	0.617	13.3	+0.32
HRSeg	PCF	91.6	1.002	0.360	21.8	+0.42
RUCNet	ML-only	85.8	1.404	0.709	14.2	+0.32
RUCNet	PCF	93.3	1.100	0.367	17.8	+0.42
U2CrackNet	ML-only	82.2	1.230	0.552	16.0	+0.43
U2CrackNet	PCF	89.3	0.849	0.266	23.1	+0.54
UNet-FocalLoss	ML-only	82.2	1.319	0.639	12.9	+0.26
UNet-FocalLoss	PCF	90.7	0.943	0.323	21.3	+0.45
SCSegamba	ML-only	98.2	1.436	0.937	4.4	+0.48
SCSegamba	PCF	97.3	1.204	0.583	16.4	+0.53
CrackFormer-II	ML-only	100.0	1.598	1.268	1.3	+0.74
CrackFormer-II	PCF	100.0	1.165	0.458	21.3	+0.53
CrackNex	ML-only	99.6	1.255	0.855	4.4	+0.52
CrackNex	PCF	97.8	1.304	0.800	13.8	+0.51
Hybrid-Segmentor	ML-only	88.4	1.355	0.828	7.1	+0.38
Hybrid-Segmentor	PCF	87.1	1.357	0.825	10.7	+0.50

## Appendix C. Measurement Agreement, All Algorithms

Figure A3 and Figure A4 give Bland–Altman agreement with the reference for all ten algorithms on both datasets. The difference is plotted against the reference rather than against the mean of the two, since the caliper and operator measurements are the reference standard rather than a second method of equal standing.

## Appendix D. Qualitative Segmentation Examples

Figure A5 and Figure A6 compare the input, ground-truth mask, ML-only mask, and PCF output for five internal samples at 0.5 m. Over-segmenting algorithms show wider masks and off-crack artifacts that are reduced by consensus fusion. More conservative masks, such as HRSeg on S2 and S5, show smaller changes, consistent with the operating-point dependence observed quantitatively.

## Appendix E. Parameter Sensitivity

Figure A7 shows the within-tolerance ROI rate and coverage across the full calibration grid for the modern-family algorithms. Both increase monotonically in the grayscale threshold and the CLAHE clip limit, with no isolated optimum, and the selected operating point (boxed) is the grid optimum in every panel.

## Appendix F. Failure Modes

Cases are selected by failure signature from the 19,098 external ROIs rather than by inspection, and from the middle of each mode’s severity distribution rather than its extreme, so each panel shows how that mode typically presents. The measurement point is marked and is required to lie on the ML-only mask, so each case is a failure of fusion rather than a mismatch between the reference location and the crack.

## Appendix G. Selection of the Width Estimator

Thirty-eight width-estimator variants spanning nine method families were evaluated on identical human-annotated masks over the same 225 field probe ROIs, so that mask quality is held constant and only the geometric estimator varies. Mean absolute error ranges from 0.466 to 1.255 mm across the variants. This comparison ranks estimators under a fixed annotation and is not a bound on achievable width accuracy: the hand-drawn masks are not used anywhere in the reported results, which compare pipeline output directly against the caliper references.

**Table A9:** Width-estimator benchmark, best variant per method family. Bold marks the variant adopted in PCF Stage 4 and its mean absolute error (MAE).

Method Family	Best Variant	MAE (mm)
Medial-axis run (adopted)	axis_run_current	**0.466**
Local axis	local_axis_r3_median	0.471
Skeleton + distance transform	skeleton_dt_local_r10_p90	0.683
Normal ray casting	normal_ray_r20	0.707
Local Feret diameter	local_feret_roi40	0.770
Contour normal	contour_normal_r20	0.803
Skeleton + DT (nearest)	skeleton_dt_nearest_r10	0.814
Multi-method consensus	multi_method_consensus	0.935
Area/length	area_over_length_roi25	1.133

*Note:* Bold identifies the estimator variant adopted in PCF Stage 4 and its corresponding MAE.

## Appendix H. Computational Cost

Table A10 reports per-ROI processing cost on the external krkCMd set. For the modern algorithms the PCF stage adds roughly 0.25 s per ROI at about 2.2–2.3 GB peak memory; the older probability-map harness (DeepLabv3+, DeepCrack, HRSeg) carries a slower full-resolution PCF path. All processing runs server-side; in the AR prototype the headset only captures and displays.

**Table A10:** Per-ROI runtime and peak memory on krkCMd. ML time is reported where the harness executes network inference per frame (probability-map models); the PaddleSeg and modern algorithms consume precomputed masks in this benchmark, so only the PCF stage is timed.

Algorithm	Params (M)	ML (s/ROI)	PCF (s/ROI)	Peak RSS (MB)
DeepLabv3+	61.0	0.89	0.24	9064
DeepCrack	30.9	0.34	0.25	8703
HRSeg	5.5	0.44	0.25	9128
RUCNet	25.5	0.93	0.25	2225
UNet-FocalLoss	13.4	0.23	0.25	2216
U2CrackNet	1.2	0.29	0.25	2222
SCSegamba	2.8	0.19	0.25	2269
CrackFormer-II	5.0	0.33	0.26	2297
Hybrid-Segmentor	237.9	1.27	0.26	2278
CrackNex	102.4	0.98	0.24	2218
